# Exploiting User Clustering and Fixed Power Allocation for Multi-Antenna UAV-Assisted IoT Systems

**DOI:** 10.3390/s23125537

**Published:** 2023-06-13

**Authors:** Sang Quang Nguyen, Anh-Tu Le, Chi-Bao Le, Phu Tran Tin, Yong-Hwa Kim

**Affiliations:** 1 Science and Technology Application for Sustainable Development Research Group, Ho Chi Minh City University of Transport, Ho Chi Minh City 700000, Vietnam; sang.nguyen@ut.edu.vn; 2 Faculty of Electrical and Electronics Engineering, Ton Duc Thang University, Ho Chi Minh City 700000, Vietnam; tule.iuh@gmail.com; 3 Faculty of Electronics Technology, Industrial University of Ho Chi Minh City (IUH), Ho Chi Minh City 700000, Vietnam; lechibao@iuh.edu.vn; 4 Data Science Laboratory, Faculty of Information Technology, Ton Duc Thang University, Ho Chi Minh City 700000, Vietnam; 5 Department of Data Science, Korea National University of Transportation (KNUT), Uiwang-si 16106, Gyeonggi-do, Republic of Korea

**Keywords:** NOMA, UAV, maximum ratio transmission, AF, outage probability, ergodic capacity

## Abstract

Internet of Things (IoT) systems cooperative with unmanned aerial vehicles (UAVs) have been put into use for more than ten years, from transportation to military surveillance, and they have been shown to be worthy of inclusion in the next wireless protocols. Therefore, this paper studies user clustering and the fixed power allocation approach by placing multi-antenna UAV-mounted relays for extended coverage areas and achieving improved performance for IoT devices. In particular, the system enables UAV-mounted relays with multiple antennas together with non-orthogonal multiple access (NOMA) to provide a potential way to enhance transmission reliability. We presented two cases of multi-antenna UAVs such as maximum ratio transmission and the best selection to highlight the benefits of the antenna-selections approach with low-cost design. In addition, the base station managed its IoT devices in practical scenarios with and without direct links. For two cases, we derive closed-form expressions of outage probability (OP) and closed-form approximation ergodic capacity (EC) generated for both devices in the main scenario. The outage and ergodic capacity performances in some scenarios are compared to confirm the benefits of the considered system. The number of antennas was found to have a crucial impact on the performances. The simulation results show that the OP for both users strongly decreases when the signal-to-noise ratio (SNR), number of antennas, and fading severity factor of Nakagami-*m* fading increase. The proposed scheme outperforms the orthogonal multiple access (OMA) scheme in outage performance for two users. The analytical results match Monte Carlo simulations to confirm the exactness of the derived expressions.

## 1. Introduction

In recent years, unmanned aerial vehicles (UAVs) have been studied and recommended for possible applications in the Internet of Things (IoT) due to their prominent features related to low-cost implementation and high flexibility [1,2,3,4,5]. As one of the effective system models, a UAV-mounted relay can be easily employed, since it provides a flexible coverage area in comparison with conventional fixed relays and increases the network capacity [6]. For UAV-enabled/assisted ground communications, a UAV relay could be a wirelessly-powered device since it harvests energy from the radio signals received from a nearby base station to improve energy efficiency (EE) [7]. The authors in [7] presented the closed-form expressions to showcase two main system performance metrics such as ergodic capacity and outage behavior by enabling both amplify-and-forward (AF) and decode-and-forward (DF) relaying protocols. In [8], the authors examined the case of the UAV carrying limited energy concerning optimal power allocation. They considered the problem of flying path optimization while the UAV is allowed to fly flexibly. To enhance energy-harvesting efficiency and data collection, the communication time and the distances between the access point and ground devices are optimized.

UAVs combined with cooperative non-orthogonal multiple access (NOMA) were considered as a further version of NOMA, which enables the simultaneous transmission among multiple links using the same frequency, can increase bandwidth utilization by carefully planning the communication resource, and increases the dependability of UAV relaying systems in emergency communications [9]. In UAV-NOMA, the far user is assisted by the near user in forwarding the signal from the base station (BS), and the forwarding process occurs in cases where the transmitted signal cannot reach the far user due to obstacles or a long-distance and poor channel condition between the BS and this user [10,11,12,13]. The near user is known as a relay in cooperative non-orthogonal multiple access (C-NOMA). Thus, the C-NOMA can cover a larger area than NOMA, and its transmission reliability is also improved.

To extend the operating areas of the UAV, one can implement a relaying network [14,15,16,17,18,19]. The relays with a single antenna have been studied by many researchers in the last decade [18,19]. Although the structure of these relays is simple, their performance is lower than the relay with multiple antennas due to its low degree of freedom [20,21]. Furthermore, multiple-antenna technology can powerfully mitigate interference [22]. In [23], the authors studied a two-user C-NOMA system in which a decode-and-forward (DF) relay was equipped with multiple antennas. In [21], a NOMA system aided by multiple-antennae two-way DF relay was investigated to boost the transmission reliability of the system. The authors derived closed-form expressions for the outage probability and diversity to show the system performance. In [24], an AF/DF relay was exploited in a C-NOMA system with two users where these two users were equipped with multiple antennas. The effect of multiple antennas on the performance of the relay was examined also. In [25], a multiple-antenna downlink NOMA system for multi-user communication is considered. The transmit power and number of feedback bits were the two main optimized parameters to exploit the potential of multiple-antenna techniques. In [26], a C-NOMA system that employed multiple-antenna two-way relays is studied. Two broadcast NOMA strategies based on multiple access and time division were proposed to enhance transmission reliability. To evaluate the system performance, closed-form expressions were derived for the outage probability and diversity order corresponding with joint antenna-and-relay selection solutions in the proposed strategies. In [27,28], a combination of the C-NOMA scheme and multiple antennas is also analyzed. In [27], the AF relay is equipped with a single antenna, while the source and users are served by multiple antennas. Two antenna-relay selection schemes, namely the maximum ratio transmission (MRT) and transmit antenna selection (TAS), were considered and compared to the random selection (RS) scheme. Based on the numerical simulation results, the performance in terms of outage probability and throughput for MRT and TAS was superior to that for RS. In [29], a full-duplex (FD) multi-antenna relay was employed in a C-NOMA system to assist the communication between BS and a set of far users. The performance of the system for the multiple-antenna relay was significantly improved as compared to that for the half-duplex relay. In [30], multiple antennas at the satellite were exploited with the C-NOMA system. The authors presented an iterative penalty function-based beamforming (BF) scheme to optimize the sum rate. The authors in [31] studied the BF schemes in the multiple-antennas system to maximize the achievable secrecy-energy efficiency. In [32], a cooperative multiple-antenna relaying system was employed where a BF was considered to minimize the total transmit power. In [33], a multiple-antennas UAV network with rate-splitting multiple access was considered. The authors designed the optimization problem to maximize the sum rate of our system. In addition, Table 1 summarizes the related work.

Although [25,26,27,28,29,30,31,32,33] studied the benefits of the multi-antenna approach to enhancing performance at destinations of a wireless system, it is still a crucial problem for in-depth analysis to provide more guidelines in the design of effective antenna selection for UAV-aided IoT systems in practice. The main contributions of this paper are summarized as follows

**Figure 1:**
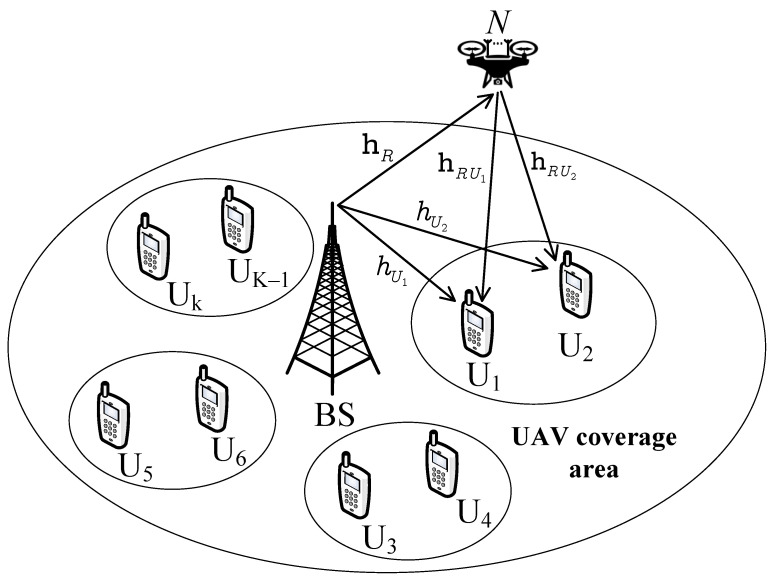
Multi-antenna UAV-aided IoT network.

**Figure 2:**
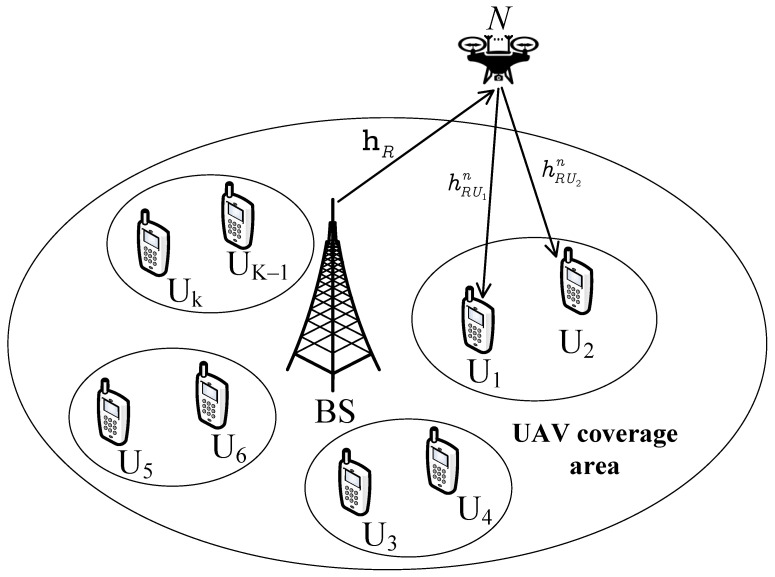
Separated antenna selection for UAV-aided IoT network.

- We provide two practical scenarios of the multi-antenna relay to evaluate a UAV-aided IoT system that can be implemented while the system still leverages the advantage of a multi-antenna UAV. Compared with the traditional multiple input multiple output (MIMO) techniques, we prefer the antenna selection approach to reduce the cost of design and the complexity of signal processing. While Figure 1 exhibits the way that the relay leverages Maximum Ratio Transmission for signal transmission, the antenna-selection approach in Figure 2 inherently enjoys the merits of reducing hardware complexity and cost.
- Nakagami-*m* fading is suitable for characterizing a wide class of fading channel conditions for UAV links. Therefore, we focus on UAVs with the analysis of the Nakagami-*m* fading model rather than Rayleigh’s fading model, which was applied in lots of previous studies. In addition, the two-user scenario of NOMA needs to evaluate the performance when the fixed power allocation is adopted. To confirm the superiority of those schemes, the closed-form expressions for the outage probability and closed-form approximation of the ergodic capacity are derived.
- The outage probability for the multiple-antenna UAV is compared in cases with/ without direct links, NOMA, and OMA to evaluate the effectiveness of the UAV equipped with multiple antennas. We investigate the impacts of the transmit signal-to-noise ratio (SNR) and the number of antennas on the outage probability and ergodic capacity. From these results, one can choose a suitable number of antennas and SNR threshold to balance the demand for performance and the cost of design.

The rest of this paper is organized as follows. Section 2 institutes the system model. In Section 3 and Section 4, the closed-form outage probability is derived for Figure 1 and Figure 2, respectively. Section 5 and Section 6 show the ergiduc capacity analysis for Figure 1 and Figure 2, respectively. Section 7 shows simulation results. Finally, conclusions are drawn in Section 8.

**Notation:** Vectors are symbolized by bold-faced letters, e.g., x,${∥.∥}_{F}$ specifies the Frobenius norm, ${\left(.\right)}^{T}$ and ${\left(.\right)}^{H}$ denote the normal and Hermitian transpose, respectively; $\mathrm{Pr}\left(.\right)$ denotes the probability operator; $E\left\{.\right\}$ is the Expectation operator; $K_{n}\left(.\right)$ is the so-called Bessel function; the probability density function (PDF) and the cumulative distribution function (CDF) of a random variable *X* are represented as $F_{X}\left(.\right)$ and $f_{X}\left(.\right)$, respectively.

## 2. System Model

We consider a downlink multiple-antenna UAV-aided IoT system, as shown in Figure 1. In this scenario, a base station (BS) wants to transfer the information to *K* users, in which two users in the group are located in separated clusters. The user $U_{k}$ could be a strong user or a weak user (k=1,2,3,…,K). The BS works with different frequencies assigned to these clusters following OMA. To evaluate the performance of a particular cluster, we refer to the strong user $U_{1}$ and the weak user $U_{2}$ with the help of an AF-based UAV (*R*), as illustrated in Figure 1. It is noted that the multiple users served by BS are divided into many groups, and each group contains two users. In addition, the BS and two users are equipped with a single antenna, and the UAV (relay) *R* is equipped with *N* antennas. In the IoT system, multiple-input multiple-output (MIMO) techniques can be employed to mitigate the detrimental effects of unavoidable fading when the base station uses multiple antennas for transmitting and receiving signals. Although using the MIMO technique could be a promising way to improve the capacity and system performance, the transceivers need multiple radio frequency (RF) chains leading to higher power consumption and higher hardware complexity. In this article, we design antenna selection (AS) schemes applied at the BS to reduce the number of RF chains and still maintain the advantages of MIMO systems. Perfect channels state information (CSI) is assumed to be available at the BS. In addition, we assume that the fading channels are distributed over Nakagami-*m* channels, which are usually characterized by channels connected to UAV.

The channel coefficients between BS and *R* are denoted by the 1×N matrix $h_{R}=\left[h_{R}^{1},h_{R}^{2},\ldots ,h_{R}^{N}\right]$ where each element $h_{R}^{n}$ with fading parameter $m_{R}$ is called independent and identically distributed (i.i.d.), and the expectation $E\left\{{\left|h_{R}^{n}\right|}^{2}\right\}=\lambda_{R}$ width $n\in \left\{1,2,\ldots ,N\right\}$. Likewise, the channel coefficients between *R* and two users $\left(U_{i}\right)$, $i\in \left\{1,2\right\}$ are denoted by the N×1 matrix $h_{RU_{i}}=\left[h_{RU_{i}}^{1},h_{RU_{i}}^{2},\ldots ,h_{RU_{i}}^{N}\right]$ in which each element $h_{RU_{i}}^{n}$ is also i.i.d. and fulfills $E\left\{{\left|h_{RU_{i}}^{n}\right|}^{2}\right\}=\lambda_{RU_{i}}$∀n∈N with fading value $m_{RU_{i}}$. Finally, the direct connection channel coefficients between BS and $U_{i}$ are given by $h_{U_{i}}∼\mathrm{Nakagami}\left(m_{U_{i}},\lambda_{U_{i}}\right)$, where $m_{U_{i}}$ is the corresponding distribution parameter and $E\left\{{\left|h_{U_{i}}\right|}^{2}\right\}=\lambda_{U_{i}}$ [39].

Following the NOMA principle and in order to provide more user fairness, we assume that ${\left|h_{U_{2}}\right|}^{2}>{\left|h_{U_{1}}\right|}^{2}$, $a_{2}>a_{1}$ with $a_{1}$ and $a_{2}$ are the power allocation coefficients and $a_{1}+a_{2}=1$ [40,41]. In this article, we just focus on the analysis of two users located in a particular cluster and assume the performance at other clusters is similar. The BS transmits the superposition coding signal, which is combined by two signals $x_{1}$ and $x_{2}$ to *R* and two users in phase 1. The expectations of signals $x_{1}$ and $x_{2}$ are assumed that $E\left[x_{1}^{2}\right]=E\left[x_{2}^{2}\right]=1$ in which $E\left[.\right]$ is called the expected operator. Hence, the transmitted signal expression at BS is given by

(1) $$\bar{x}=\sum_{i=1}^{2}\sqrt{a_{i}P_{S}}x_{i},$$

where $x_{1}$ and $x_{2}$ are the messages for $U_{1}$ and $U_{2}$, respectively. We assume that $n_{R}$, $n_{RU_{i}}$, $n_{U_{i}}$ denotes the additive white Gaussian noise (AWGN) with mean power $N_{0}$.

## 3. The Outage Performance of Figure 1

The received signals at $U_{1}$, $U_{2}$ and *R* are, respectively, given by

(2) $$\begin{array}{cc} \begin{array}{cc} y_{U_{i}}= & h_{U_{i}}\bar{x}+n_{U_{i}}\\ = & h_{U_{i}}\left(\sqrt{a_{1}P_{S}}x_{1}+\sqrt{a_{2}P_{S}}x_{2}\right)+n_{U_{i}} \end{array} & \begin{array}{c} i\in \left\{1,2\right\} \end{array}, \end{array}$$

and

(3) $$\begin{array}{cc} y_{R}= & h_{R}v_{R}^{H}\bar{x}+n_{R}\\ = & \frac{h_{R}h_{R}^{H}}{{∥h_{R}∥}_{F}}\left(\sqrt{a_{1}P_{S}}x_{1}+\sqrt{a_{2}P_{S}}x_{2}\right)+n_{R}, \end{array}$$

where $P_{S}$ is the transmit power and $v_{R}\in C{}^{N\times 1}$ is the receive beamforming vector at the *R*. By employing the maximum ratio combining (MRC) scheme, each user $U_{1}$ and $U_{2}$ employs the normalized vector $v_{R}$ as $v_{R}=\frac{h_{R}^{H}}{{∥h_{R}∥}_{F}}$ in [42].

In the second phase, *R* transmits the signal $x_{R}=\bar{G}y_{R}$ to both $U_{1}$ and $U_{2}$, where $\bar{G}$ denotes the amplifying gain at relay, i.e.,

(4) $$\bar{G}=\sqrt{\frac{P_{R}}{P_{R}{∥h_{R}∥}^{2}+N_{0}}},$$

where $P_{R}$ denotes the transmit power of the relay. Without loss of generality, we assume that the transmit power at R is equal to the transmit power of the BS, i.e., $P_{R}=P_{S}=P$. Therefore, the received signals at $U_{i}$ (forwarded by *R*) are given by [43]:

(5) $$\begin{array}{cc} \begin{array}{cc} y_{RU_{i}}= & h_{RU_{i}}k_{i}x_{R}+n_{RU_{i}}\\ = & \bar{G}h_{RU_{i}}k_{i}\frac{h_{R}h_{R}^{H}}{{∥h_{R}∥}_{F}}\sqrt{a_{1}P_{S}}x_{1}\\ \; & +\bar{G}h_{RU_{i}}k_{i}\frac{h_{R_{n}}h_{R}^{H}}{{∥h_{R}∥}_{F}}\sqrt{a_{2}P_{S}}x_{2}\\ \; & +\bar{G}h_{RU_{i}}k_{i}n_{R}+n_{RU_{i}}. \end{array} & ,i\in \left\{1,2\right\} \end{array}$$

By employing the Maximum Ratio Transmission (MRT) scheme, the relay obtains the following beamforming vector $k_{i}\in {}^{N\times 1}$ to steer the signal in the direction of two users $U_{1}$ and $U_{2}$ with $k_{i}=\frac{{\left(h_{RU_{i}}^{T}\right)}^{H}}{{∥h_{RU_{i}}^{T}∥}_{F}}$ in [42,44].

To calculate the instantaneous signal-to-interference-plus-noise ratio (SINR) of the two phases, let us define the average transmit SNR, $\rho =\frac{P}{N_{0}}$ and the random variables (RVs) $X_{i}={\left|h_{U_{i}}\right|}^{2}$, where $Y={∥h_{R}∥}_{F}^{2}$ and $Z_{i}={∥h_{RU_{i}}∥}_{F}^{2}$ represent the instantaneous SNRs of the links $S\to U_{i}$, S→R and $R\to U_{i}$, respectively.

There are two phases of signal processing in the NOMA system. In the first phase, other signal components are treated as interference by $U_{2}$ while decoding their own message $x_{2}$. The SINR is used to decode the signal $x_{2}$ with a direct link given by

(6) $$\gamma_{U_{2}}=\frac{a_{2}\rho X_{2}}{a_{1}\rho X_{2}+1}.$$

Similarly, the instantaneous SINR at $U_{1}$ to detect $x_{2}$ is given as

(7) $$\gamma_{U_{2}\to U_{1}}=\frac{a_{2}\rho X_{1}}{a_{1}\rho X_{1}+1}.$$

After SIC, we assume the perfect SIC is at the receiver side. Therefore, the received SNR at $U_{1}$ to detect its own message $x_{1}$ is written as

(8) $$\gamma_{U_{1}}=a_{1}\rho X_{1}.$$

In the second phase, the instantaneous SINR at $U_{2}$ relating to link $R\to U_{2}$ is calculated by applying the same procedure as the first phase and is thus given by

(9) $$\gamma_{RU_{2},x_{2}}=\frac{a_{2}\rho^{2}YZ_{2}}{a_{1}\rho^{2}YZ_{2}+\rho Y+\rho Z_{2}+1}.$$

Considering the link $R\to U_{1}$, the instantaneous SINR at $U_{1}$ to detect $x_{2}$ and the instantaneous SNR at $U_{1}$ to detect its own data $x_{1}$ are, respectively, given by

(10) $$\gamma_{RU_{1},x_{2}}=\frac{a_{2}\rho^{2}YZ_{1}}{a_{1}\rho^{2}YZ_{1}+\rho Y+\rho Z_{1}+1},$$

and

(11) $$\gamma_{RU_{1},x_{1}}=\frac{a_{1}\rho^{2}YZ_{1}}{\rho Y+\rho Z_{1}+1}.$$

Based on the selection combination, the instantaneous SINRs at $U_{2}$ and $U_{1}$ could be given as [45]

(12a) $$\begin{array}{cc} \; & {\bar{\gamma }}_{U_{2}}=\max\left(\gamma_{U_{2}},\gamma_{RU_{2},x_{2}}\right), \end{array}$$

(12b) $$\begin{array}{cc} \; & {\bar{\gamma }}_{U_{1}}=\max\left(\gamma_{U_{1}},\gamma_{RU_{1},x_{1}}\right). \end{array}$$

Based on the quality of service of two users, their target SINRs can be determined. Each user has its own the SNR threshold, $\varepsilon_{th_{i}}=2^{2R_{i}}-1,i\in \left\{1,2\right\}$ where $R_{1}$ is the target rate at $U_{1}$ to detect $x_{1}$ and $R_{2}$ is the target rate at $U_{1}$ to detect $x_{2}$. For simplicity, we assume the SNR thresholds of $U_{2}$ and $U_{1}$ are both equal, i.e., $\varepsilon_{th_{1}}=\varepsilon_{th_{2}}=\varepsilon_{th}$.

### 3.1. Outage Probability at $U_{2}$

According to [46,47], the cumulative distribution functions (CDF) of the RVs $X_{i}$, *Y* and $Z_{i}$, respectively, are given by

(13a) $$\;F_{X_{i}}\left(x\right)=1-e^{-\mu_{U_{i}}x}\sum_{t=0}^{m_{U_{i}}-1}\frac{\mu_{U_{i}}^{t}x^{t}}{t!},i\in \left\{1,2\right\},$$

(13b) $$F_{Y}\left(x\right)=1-e^{-\mu_{R}x}\sum_{t=0}^{m_{R}N-1}\frac{\mu_{R}^{t}x^{t}}{t!},$$

(13c) $$\;F_{Z_{i}}\left(x\right)=1-e^{-\mu_{RU_{i}}x}\sum_{t=0}^{m_{RU_{i}}N-1}\frac{\mu_{RU_{i}}^{t}x^{t}}{t!},i\in \left\{1,2\right\},$$

and the probability density function (PDF) of the RVs $X_{i}$, *Y* and $Z_{i}$, respectively, are given by

(14a) $$\;f_{X_{i}}\left(x\right)=\frac{\mu_{U_{i}}^{m_{U_{i}}}x^{m_{U_{i}}-1}}{\Gamma \left(m_{U_{i}}\right)}e^{-\mu_{U_{i}}x},i\in \left\{1,2\right\},$$

(14b) $$f_{Y}\left(x\right)=\mu_{R}^{m_{R}N}\frac{x^{m_{R}N-1}}{\Gamma \left(m_{R}N\right)}e^{-\mu_{R}x},$$

(14c) $$\;f_{Z_{i}}\left(x\right)=\frac{\mu_{RU_{i}}^{m_{RU_{i}}N}x^{m_{RU_{i}}N-1}e^{-\mu_{RU_{i}}x}}{\Gamma \left(m_{RU_{i}}N\right)},i\in \left\{1,2\right\},$$

where $\mu_{U_{i}}=\frac{m_{U_{i}}}{\lambda_{U_{i}}}$, $\mu_{R}=\frac{m_{R}}{\lambda_{R}}$ and $\mu_{RU_{i}}=\frac{m_{RU_{i}}}{\lambda_{RU_{i}}}$ are the parameters of multipath fading associated with ${\left|h_{U_{i}}\right|}^{2}$, ${∥h_{R}∥}_{F}^{2}$ and ${∥h_{RU_{i}}∥}_{F}^{2}$, respectively.

Then, the outage probability at $U_{2}$ can be given by

(15) $$\begin{array}{cc} {\bar{P}}_{2}^{I}= & \mathrm{Pr}\left({\bar{\gamma }}_{U_{2}}<\varepsilon_{th}\right)\\ = & \mathrm{Pr}\left(\max\left(\gamma_{U_{2}},\gamma_{RU_{2},x_{2}}\right)<\varepsilon_{th}\right)\\ = & {\bar{A}}_{1}\times {\bar{A}}_{2}. \end{array}$$

where ${\bar{A}}_{1}=\mathrm{Pr}\left(\gamma_{U_{2}}<\varepsilon_{th}\right)$ and ${\bar{A}}_{2}=\mathrm{Pr}\left(\gamma_{RU_{2},x_{2}}<\varepsilon_{th}\right)$.

**Proposition** **1.** *The closed-form expression of the outage probability at user $U_{2}$ can be given by*

(16) $$\begin{array}{cc} {\bar{P}}_{2}^{I}= & \left(1-e^{-\mu_{U_{2}}\theta }\sum_{a=0}^{m_{U_{2}}-1}\frac{\mu_{U_{2}}^{a}\theta^{a}}{a!}\right)\left[1-2\sum_{b=0}^{m_{R}N-1}\sum_{q=0}^{b}\sum_{n=0}^{m_{RU_{2}}N-1}\right.\\ \; & \times 2\left(\begin{array}{c} b\\ q \end{array}\right)\left(\begin{array}{c} m_{RU_{2}}N-1\\ n \end{array}\right)\frac{{\left(\theta^{2}+\theta \rho^{-1}\right)}^{q}e^{-\theta \left(\mu_{RU_{2}}+\mu_{R}\right)}}{b!\Gamma \left(m_{RU_{2}}N\right)}\\ \; & \times \mu_{R}^{b}\theta^{m_{RU_{2}}N+b-q-n-1}{\left(\frac{\mu_{R}\left(\theta^{2}+\theta \rho^{-1}\right)}{\mu_{RU_{2}}}\right)}^{\frac{n-q+1}{2}}\\ \; & \left.\times \mu_{RU_{2}}^{m_{RU_{2}}N}K_{n-q+1}\left(2\sqrt{\mu_{R}\mu_{RU_{2}}\left(\theta^{2}+\theta \rho^{-1}\right)}\right)\right]. \end{array}$$

*where $\theta =\frac{\varepsilon_{th}}{\rho \left(a_{2}-a_{1}\varepsilon_{th}\right)}$.*

Proof of Proposition 1, see Appendix A.

### 3.2. Outage Probability at $U_{1}$

Similarly, the outage probability at user $U_{1}$ can be computed by

(17) $$\begin{array}{cc} {\bar{P}}_{1}^{I}= & \mathrm{Pr}\left({\bar{\gamma }}_{U_{1}}<\varepsilon_{th}\right)\\ = & \mathrm{Pr}\left(\max\left(\gamma_{U_{1}},\gamma_{RU_{1},x_{1}}\right)<\varepsilon_{th}\right)\\ = & \underset{{\bar{B}}_{1}}{\underset{︸}{\mathrm{Pr}\left(\gamma_{U_{1}}<\varepsilon_{th}\right)}}\times \underset{{\bar{B}}_{2}}{\underset{︸}{\mathrm{Pr}\left(\gamma_{RU_{1},x_{1}}<\varepsilon_{th}\right)}} \end{array}$$

**Proposition** **2.** *The closed-form expression of the outage probability at user $U_{1}$ can be computed by*

(18) $$\begin{array}{cc} {\bar{P}}_{1}^{I}= & \left(1-e^{-\mu_{U_{1}}\varphi }\sum_{t=0}^{m_{U_{1}}-1}\frac{\mu_{U_{1}}^{t}\varphi^{t}}{t!}\right)\left[1-2\sum_{k=0}^{m_{R}N-1}\sum_{r=0}^{k}\sum_{d=0}^{m_{RU_{1}}N-1}\right.\\ \; & \times \left(\begin{array}{c} k\\ r \end{array}\right)\left(\begin{array}{c} m_{RU_{1}}N-1\\ d \end{array}\right)\frac{{\left(\varphi +\rho^{-1}\right)}^{r}e^{-\varphi \left(\mu_{RU_{1}}+\mu_{R}\right)}}{k!\Gamma \left(m_{RU_{1}}N\right)}\\ \; & \times \mu_{R}^{k}\varphi^{m_{RU_{1}}N+k-d-1}{\left(\frac{\mu_{R}\left(\varphi^{2}+\varphi \rho^{-1}\right)}{\mu_{RU_{1}}}\right)}^{\frac{d-r+1}{2}}\\ \; & \left.\times \mu_{RU_{1}}^{m_{RU_{1}}N}K_{d-r+1}\left(2\sqrt{\mu_{R}\mu_{RU_{1}}\varphi^{2}+\varphi \rho^{-1}}\right)\right], \end{array}$$

*where $\varphi =\frac{\varepsilon_{th}}{a_{1}\rho }$*

**Proof.**  Here, ${\bar{B}}_{1}$ is calculated as

(19) $$\begin{array}{cc} {\bar{B}}_{1}= & \mathrm{Pr}\left(\gamma_{U_{1}}<\varepsilon_{th}\right)\\ = & \mathrm{Pr}\left(X_{1}<\frac{\varepsilon_{th}}{a_{1}\rho }\right)\\ = & F_{X_{1}}\left(\frac{\varepsilon_{th}}{a_{1}\rho }\right)\\ = & 1-e^{-\mu_{U_{1}}\varphi }\sum_{t=0}^{m_{U_{1}}-1}\frac{\mu_{U_{1}}^{t}\varphi^{t}}{t!}. \end{array}$$

Next, ${\bar{B}}_{2}$ is calculated as

(20) $$\begin{array}{cc} {\bar{B}}_{2}= & \mathrm{Pr}\left(\gamma_{RU_{1},x_{1}}<\varepsilon_{th}\right)\\ = & 1-\mathrm{Pr}\left(Y>\frac{\varphi \left(Z_{1}+\rho^{-1}\right)}{Z_{1}-\varphi },Z_{1}>\varphi \right)\\ = & 1-\int_{\varphi }^{\infty }f_{Z_{1}}\left(x\right)\left[1-F_{Y}\left(\frac{\varphi \left(x+\rho^{-1}\right)}{x-\varphi }\right)\right]dx\\ = & 1-\sum_{k=0}^{m_{R}N-1}\frac{\mu_{RU_{1}}^{m_{RU_{1}}N}\mu_{R}^{k}}{k!\Gamma \left(m_{RU_{1}}N\right)}\int_{\varphi }^{\infty }x^{m_{RU_{1}}N-1}e^{-\mu_{RU_{1}}x}\\ \; & \times e^{-\frac{\mu_{R}\varphi \left(x+\rho^{-1}\right)}{x-\varphi }}{\left(\frac{\varphi \left(x+\rho^{-1}\right)}{x-\varphi }\right)}^{k}dx. \end{array}$$

Let t=x−φ→t+φ=x→dt=dx, ${\bar{B}}_{2}$ be given as

(21) $$\begin{array}{cc} {\bar{B}}_{2}= & 1-\sum_{k=0}^{m_{R}N-1}\frac{\mu_{RU_{1}}^{m_{RU_{1}}N}\mu_{R}^{k}}{k!\Gamma \left(m_{RU_{1}}N\right)}\int_{0}^{\infty }{\left(t+\varphi \right)}^{m_{RU_{1}}N-1}e^{-\mu_{RU_{1}}\left(t+\varphi \right)}\\ \; & \times e^{-\frac{\mu_{R}\varphi \left(t+\varphi +\rho^{-1}\right)}{t}}{\left(\frac{\varphi \left(t+\varphi +\rho^{-1}\right)}{t}\right)}^{k}dt\\ = & 1-\sum_{k=0}^{m_{R}N-1}\frac{\mu_{RU_{1}}^{m_{RU_{1}}N}\mu_{R}^{k}e^{-\varphi \left(\mu_{RU_{1}}+\mu_{R}\right)}}{k!\Gamma \left(m_{RU_{1}}N\right)}\\ \; & \times \int_{0}^{\infty }{\left(t+\varphi \right)}^{m_{RU_{1}}N-1}e^{-\frac{\mu_{R}\varphi \left(\varphi +\rho^{-1}\right)}{t}-\mu_{RU_{1}}t}\\ \; & \times {\left(\frac{\varphi \left(t+\varphi +\rho^{-1}\right)}{t}\right)}^{k}dt. \end{array}$$

With the help of Equations (1.111), (3.471.9) [48] and after some manipulations, we have

(22) $$\begin{array}{cc} {\bar{B}}_{2}= & 1-2\sum_{k=0}^{m_{R}N-1}\sum_{r=0}^{k}\sum_{d=0}^{m_{RU_{1}}N-1}\left(\begin{array}{c} k\\ r \end{array}\right)\left(\begin{array}{c} m_{RU_{1}}N-1\\ d \end{array}\right)\\ \; & \times \frac{\mu_{R}^{k}\varphi^{m_{RU_{1}}N+k-d-1}e^{-\varphi \left(\mu_{RU_{1}}+\mu_{R}\right)}}{k!\Gamma \left(m_{RU_{1}}N\right)}\\ \; & \times \mu_{RU_{1}}^{m_{RU_{1}}N}{\left(\varphi +\rho^{-1}\right)}^{r}{\left(\frac{\mu_{R}\left(\varphi^{2}+\varphi \rho^{-1}\right)}{\mu_{RU_{1}}}\right)}^{\frac{d-r+1}{2}}\\ \; & \times K_{d-r+1}\left(2\sqrt{\mu_{R}\mu_{RU_{1}}\varphi^{2}+\varphi \rho^{-1}}\right). \end{array}$$

Substituting (22) and (19) into (17), the expression of (18) can be obtained. □

**Remark** **1.** *Although derivations of outage behavior are complicated, we still realize that channel parameters and the number of transmit antennas can be the main factors affecting the system performance. For example: in (18), the outage behavior relies on m, N.*

## 4. The Outage Performance of Figure 2: Separated Antenna Selection Approach

**Figure 3:**
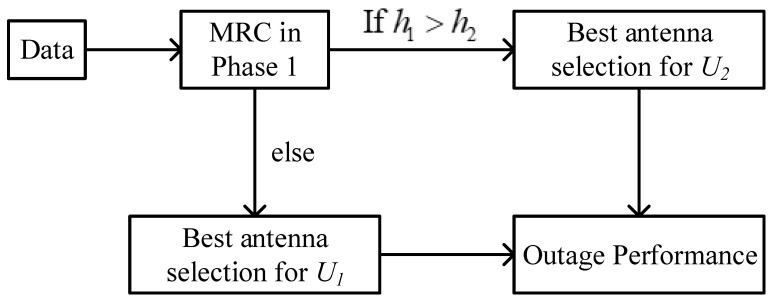
The block diagram of the antenna selection for Figure 2.

In Figure 2, each user follows the antenna selection scheme itself. This way, the performance of each user can be maximized as expected. We will present how Figure 2 is different from Figure 1 in terms of outage performance. In addition, Figure 3 shows the block diagram of the antenna selection for Figure 2. We assume the MRC method from BS to R and the optimal antenna from R to two users by the transmit antenna selection method.

By employing the antenna-selection technique for separated users, the received signal at each $U_{i}$ is first written as

(23) $$\begin{array}{cc} \begin{array}{cc} y_{RU_{i}}^{n}= & \bar{G}h_{RU_{i}}^{n}y_{R}+n_{RU_{i}}^{n}\\ = & \bar{G}h_{RU_{i}}^{n}\frac{h_{R}h_{R}^{H}}{{∥h_{R}∥}_{F}}\sqrt{a_{1}P_{S}}x_{1}\\ \; & +\bar{G}h_{RU_{i}}^{n}\frac{h_{R_{n}}h_{R}^{H}}{{∥h_{R}∥}_{F}}\sqrt{a_{2}P_{S}}x_{2}\\ \; & +\bar{G}h_{RU_{i}}^{n}n_{R}+n_{RU_{i}}^{n}. \end{array} & ,i\in \left(1,2\right) \end{array}$$

### 4.1. Best Antenna Serving $U_{1}$

In this case, the BS selects the ideal antenna to obtain the highest performance at $U_{1}$. When $U_{2}$ performance is assured owing to greater normalized channel gain and the source chooses an appropriate antenna to serve $U_{1}$, this method can maximize the system’s performance. The chosen antenna, represented by $n_{1}^{*}$, may therefore be written as

(24) $$n_{1}^{*}=\arg\max_{n=1,2,\ldots ,N}{\left|h_{RU_{1}}^{n}\right|}^{2}.$$

According to (23), the equivalent instantaneous end-to-end SINR of $U_{1}$ can be written as

(25) $${\bar{\gamma }}_{RU_{1},x_{2}}^{n^{*}}=\frac{a_{2}\rho^{2}Y{\bar{Z}}_{1}^{n^{*}}}{a_{1}\rho^{2}Y{\bar{Z}}_{1}^{n^{*}}+\rho Y+\rho {\bar{Z}}_{1}^{n^{*}}+1},$$

and

(26) $${\bar{\gamma }}_{RU_{1},x_{1}}^{n^{*}}=\frac{a_{1}\rho^{2}Y{\bar{Z}}_{1}^{n^{*}}}{\rho Y+\rho {\bar{Z}}_{1}^{n^{*}}+1},$$

where ${\bar{Z}}_{i}^{n^{*}}={\left|h_{RU_{i}}^{n^{*}}\right|}^{2}$ with $i\in \left\{1,2\right\}$.

In addition, the PDF and CDF of ${\bar{Z}}_{i}^{n^{*}}$ can be re-expressed as

(27) $$\begin{array}{cc} \; & f_{{\bar{Z}}_{i}^{n^{*}}}\left(x\right)=\frac{N\mu_{RU_{i}}^{m_{RU_{i}}}x^{m_{RU_{i}}-1}e^{-\mu_{RU_{i}}x}}{\left(m_{RU_{i}}-1\right)!}\\ \; & \times {\left(1-e^{-\mu_{RU_{i}}x}\sum_{t=0}^{m_{RU_{i}}-1}\frac{\mu_{RU_{i}}^{t}x^{t}}{t!}\right)}^{N-1}\\ \; & =\frac{N\mu_{RU_{i}}^{m_{RU_{i}}}x^{m_{RU_{i}}-1}e^{-\mu_{RU_{i}}x}}{\left(m_{RU_{i}}-1\right)!}\\ \; & \times \sum_{a=0}^{N-1}\left(\begin{array}{c} N-1\\ a \end{array}\right){\left(-1\right)}^{a}e^{-\mu_{RU_{i}}ax}\sum_{j_{0}+\ldots +j_{m_{RU_{i}}-1}=a}\\ \; & \times {\left(\begin{array}{c} a\\ j_{0},\ldots ,j_{m_{RU_{i}}-1} \end{array}\right)\prod_{b=0}^{m_{RU_{i}}-1}\left(\frac{\mu_{RU_{i}}^{b}x^{b}}{b!}\right)}^{j_{b}}, \end{array}$$

and

(28) $$\begin{array}{cc} \; & F_{{\bar{Z}}_{i}^{n^{*}}}\left(x\right)={\left(1-e^{-\mu_{RU_{i}}x}\sum_{t=0}^{m_{RU_{i}}-1}\frac{\mu_{RU_{i}}^{t}x^{t}}{t!}\right)}^{N}\\ \; & =\sum_{a=0}^{N}\left(\begin{array}{c} N\\ a \end{array}\right){\left(-1\right)}^{a}e^{-\mu_{RU_{i}}ax}\sum_{j_{0}+\ldots +j_{m_{RU_{i}}-1}=a}\\ \; & \times {\left(\begin{array}{c} a\\ j_{0},\ldots ,j_{m_{RU_{i}}-1} \end{array}\right)\prod_{b=0}^{m_{RU_{i}}-1}\left(\frac{\mu_{RU_{i}}^{b}x^{b}}{b!}\right)}^{j_{b}}. \end{array}$$

From (25) and (26), we can write the expression of $P_{1}^{II}$ as in (29)

(29) $$\begin{array}{c} {\bar{P}}_{1}^{II}=\mathrm{Pr}\left(\min\left({\bar{\gamma }}_{RU_{1},x_{2}}^{n^{*}},{\bar{\gamma }}_{RU_{1},x_{1}}^{n^{*}}\right)<\varepsilon_{th}\right)\\ =1-\mathrm{Pr}\left({\bar{\gamma }}_{RU_{1},x_{2}}^{n^{*}}>\varepsilon_{th},{\bar{\gamma }}_{RU_{1},x_{1}}^{n^{*}}>\varepsilon_{th}\right)\\ =1-\mathrm{Pr}\left(\begin{array}{c} \frac{a_{2}\rho^{2}Y{\bar{Z}}_{1}^{n^{*}}}{a_{1}\rho^{2}Y{\bar{Z}}_{1}^{n^{*}}+\rho Y+\rho {\bar{Z}}_{1}^{n^{*}}+1}>\varepsilon_{th},\\ \frac{a_{1}\rho^{2}Y{\bar{Z}}_{1}^{n^{*}}}{\rho Y+\rho {\bar{Z}}_{1}^{n^{*}}+1}>\varepsilon_{th} \end{array}\right)\\ =1-\mathrm{Pr}\left(\begin{array}{c} Y{\bar{Z}}_{1}^{n^{*}}>\frac{\varepsilon_{th}}{\rho^{2}\left(a_{2}-\varepsilon_{th}a_{1}\right)}\left(\rho Y+\rho {\bar{Z}}_{1}^{n^{*}}+1\right),\\ Y{\bar{Z}}_{1}^{n^{*}}>\frac{\varepsilon_{th}}{a_{1}\rho^{2}}\left(\rho Y+\rho {\bar{Z}}_{1}^{n^{*}}+1\right) \end{array}\right)\\ =1-\mathrm{Pr}\left(Y{\bar{Z}}_{1}^{n^{*}}>\theta_{\max}\left(\rho Y+\rho {\bar{Z}}_{1}^{n^{*}}+1\right)\right) \end{array}$$

where $\theta_{1}=\frac{\varepsilon_{th}}{a_{1}\rho^{2}}$, $\theta_{2}=\frac{\varepsilon_{th}}{\rho^{2}\left(a_{2}-\varepsilon_{th}a_{1}\right)}$ and $\theta_{\max}=\max\left(\theta_{1},\theta_{2}\right)$.

**Proposition** **3.** *The approximated closed-form expressions of the outage probability for $U_{2}$ with Figure 2 is given by*

(30) $$\begin{array}{c} {\bar{P}}_{1}^{II}=1-\frac{\mu_{R}^{m_{R}N}e^{-\mu_{R}\theta_{\max}\rho }}{\Gamma \left(m_{R}N\right)}\sum_{q=0}^{m_{R}N-1}\left(\begin{array}{c} m_{R}N-1\\ q \end{array}\right)\\ \times {\left(\theta_{\max}\rho \right)}^{m_{R}N-q-1}\left[\frac{q!}{\mu_{R}^{q+1}}-\sum_{a=0}^{N}\left(\begin{array}{c} N\\ a \end{array}\right){\left(-1\right)}^{a}\right.\\ \times e^{-\mu_{RU_{1}}a\theta_{\max}\rho }\sum_{j_{0}+\ldots +j_{m_{RU_{1}}-1}=a}\left(\begin{array}{c} a\\ j_{0},\ldots ,j_{m_{RU_{1}}-1} \end{array}\right)\\ \times \prod_{b=0}^{m_{RU_{1}}-1}{\left(\frac{\mu_{RU_{1}}^{b}}{b!}\right)}^{j_{b}}\sum_{c=0}^{bj_{b}}\left(\begin{array}{c} bj_{b}\\ c \end{array}\right){\left[\theta_{\max}\left(\theta_{\max}\rho^{2}+1\right)\right]}^{c}\\ \times {\left(\theta_{\max}\rho \right)}^{bj_{b}-c}2{\left(\frac{\mu_{RU_{1}}a\theta_{\max}\left(\theta_{\max}\rho^{2}+1\right)}{\mu_{R}}\right)}^{\frac{q-c+1}{2}}\\ \left.\times K_{q-c+1}\left(2\sqrt{\mu_{R}\mu_{RU_{1}}a\theta_{\max}\left(\theta_{\max}\rho^{2}+1\right)}\right)\right]. \end{array}$$

Proof of Proposition 3, see Appendix B.

### 4.2. Best Antenna Serving $U_{2}$

The source BS will select the ideal antenna in Figure 2 to get the highest performance dedicated to $U_{2}$. When the $U_{1}$ performance is uncertain owing to high interference levels and/or unfavorable channel conditions, this method can maximize the system’s performance. The source then favors a suitable antenna to serve $U_{2}$. The chosen antenna, represented by $n_{2}^{*}$, may be written as

(31) $$n_{2}^{*}=\arg\max_{n=1,2,\ldots ,N}{\left|h_{RU_{2}}^{n}\right|}^{2}.$$

Thus, the received SINR at $U_{2}$ to detect $x_{2}$ is given by

(32) $${\bar{\gamma }}_{RU_{2},x_{2}}^{n^{*}}=\frac{a_{2}\rho^{2}Y{\bar{Z}}_{2}^{n^{*}}}{a_{1}\rho^{2}Y{\bar{Z}}_{2}^{n^{*}}+\rho Y+\rho {\bar{Z}}_{2}^{n^{*}}+1}.$$

The outage probability of $U_{2}$ for AF-NOMA is

(33) $$\begin{array}{cc} {\bar{P}}_{2}^{II}= & 1-\mathrm{Pr}\left({\bar{\gamma }}_{RU_{2},x_{2}}^{n^{*}}>\varepsilon_{th}\right)\\ = & 1-\mathrm{Pr}\left(\frac{a_{2}\rho^{2}Y{\bar{Z}}_{2}^{n^{*}}}{a_{1}\rho^{2}Y{\bar{Z}}_{2}^{n^{*}}+\rho Y+\rho {\bar{Z}}_{2}^{n^{*}}+1}>\varepsilon_{th}\right)\\ = & 1-\mathrm{Pr}\left(Y{\bar{Z}}_{2}^{n^{*}}>\theta_{2}\left(\rho Y+\rho {\bar{Z}}_{2}^{n^{*}}+1\right)\right). \end{array}$$

Similarly, by solving $P_{1}^{II}$, $P_{2}^{II}$ can be obtained as

(34) $$\begin{array}{cc} {\bar{P}}_{2}^{II}= & 1-\frac{\mu_{R}^{m_{R}N}e^{-\mu_{R}\theta_{2}\rho }}{\Gamma \left(m_{R}N\right)}\sum_{q=0}^{m_{R}N-1}\left(\begin{array}{c} m_{R}N-1\\ q \end{array}\right)\\ \; & \times {\left(\theta_{2}\rho \right)}^{m_{R}N-q-1}\left[\frac{q!}{\mu_{R}^{q+1}}-\sum_{a=0}^{N}\left(\begin{array}{c} N\\ a \end{array}\right){\left(-1\right)}^{a}\right.\\ \; & \times e^{-\mu_{RU_{2}}a\theta_{2}\rho }\sum_{j_{0}+\ldots +j_{m_{RU_{2}}-1}=a}\left(\begin{array}{c} a\\ j_{0},\ldots ,j_{m_{RU_{2}}-1} \end{array}\right)\\ \; & \times \prod_{b=0}^{m_{RU_{2}}-1}{\left(\frac{\mu_{RU_{2}}^{b}}{b!}\right)}^{j_{b}}\sum_{c=0}^{bj_{b}}\left(\begin{array}{c} bj_{b}\\ c \end{array}\right){\left[\theta_{2}\left(\theta_{2}\rho^{2}+1\right)\right]}^{c}\\ \; & \times {\left(\theta_{2}\rho \right)}^{bj_{b}-c}2{\left(\frac{\mu_{RU_{2}}a\theta_{2}\left(\theta_{2}\rho^{2}+1\right)}{\mu_{R}}\right)}^{\frac{q-c+1}{2}}\\ \; & \left.\times K_{q-c+1}\left(2\sqrt{\mu_{R}\mu_{RU_{2}}a\theta_{2}\left(\theta_{2}\rho^{2}+1\right)}\right)\right]. \end{array}$$

Although the design of Figure 2 costs less, the system working with Figure 1 still provides higher diversity characterization. We pay attention to examining other system performance metrics, i.e., ergodic capacity.

## 5. Figure 1: Ergodic Capacity Analysis

We first define the EC of $U_{2}$ below

(35) $$\bar{C_{2}}=E\left\{\frac{1}{2}\log_{2}\left(1+\max\left(\gamma_{U_{2}},\gamma_{RU_{2},x_{2}}\right)\right)\right\}.$$

**Proposition** **4.** *The approximated closed-form expressions of the EC for $U_{2}$ are given by
*

(36) $$\begin{array}{cc} {\bar{C}}_{2}= & \frac{1}{2\ln2}\left\{\sum_{a=0}^{m_{U_{2}}-1}\frac{\mu_{U_{2}}^{a}}{a!\rho^{a}}\left[G_{1,2}^{2,1}\left(\frac{\mu_{U_{2}}}{\rho \left(a_{2}+a_{1}\right)}\left|\begin{array}{c} 1-a-1,-\\ 1-a-1,0 \end{array}\right.\right)\right.\right.\\ \; & \times \left.\frac{1}{{\left(a_{2}+a_{1}\right)}^{a}}-\frac{1}{a_{1}^{a}}G_{1,2}^{2,1}\left(\frac{\mu_{U_{2}}}{\rho a_{1}^{a}}\left|\begin{array}{c} 1-a-1,-\\ 1-a-1,0 \end{array}\right.\right)\right]\\ \; & +\frac{\pi^{2}}{2D}\sum_{b=0}^{m_{R}N-1}\sum_{q=0}^{b}\sum_{n=0}^{m_{RU_{2}}N-1}\sum_{d=1}^{D}\left(\begin{array}{c} b\\ q \end{array}\right)\\ \; & \times \left(\begin{array}{c} m_{RU_{2}}N-1\\ n \end{array}\right)\frac{\mu_{RU_{2}}^{m_{RU_{2}}N}\mu_{R}^{b}\sqrt{1-\varphi_{d}^{2}}}{b!\Gamma \left(m_{RU_{2}}N\right)}\\ \; & \times \left(\frac{1}{\Lambda \left(\varphi_{d}\right)+{\left(a_{2}+a_{1}\right)}^{-1}}-\frac{1}{\Lambda \left(\varphi_{d}\right)+a_{1}^{-1}}\right)\Phi {\left(\varphi_{d}\right)}^{q}\\ \; & \times \sec^{2}\left(\frac{\pi \left(\varphi_{d}+1\right)}{4}\right)\left(1-e^{-\frac{\mu_{U_{2}}\Lambda \left(\varphi_{d}\right)}{\rho }}\sum_{a=0}^{m_{U_{2}}-1}\frac{\mu_{U_{2}}^{a}\Lambda {\left(\varphi_{d}\right)}^{a}}{a!\rho^{a}}\right)\\ \; & \times {\left(\frac{\Lambda \left(\varphi_{d}\right)}{\rho }\right)}^{m_{RU_{2}}N+b-q-n-1}e^{-\frac{\Lambda \left(\varphi_{d}\right)\left(\mu_{RU_{2}}+\mu_{R}\right)}{\rho }}\\ \; & \left.\times {\left(\frac{\mu_{R}\Phi \left(\varphi_{d}\right)}{\mu_{RU_{2}}}\right)}^{\frac{n-q+1}{2}}K_{n-q+1}\left(2\sqrt{\mu_{R}\mu_{RU_{2}}\Phi \left(\varphi_{d}\right)}\right)\right\}, \end{array}$$

*where $\varphi_{d}=\cos\left(\frac{2d-1}{2D}\pi \right)$, $\Lambda \left(r\right)=\tan\left(\frac{\pi \left(r+1\right)}{4}\right)$ and $\Phi \left(r\right)=\frac{\Lambda \left(r\right)}{\rho^{2}}\left(\Lambda \left(r\right)+1\right)$.*

Proof of Proposition 4, see Appendix C.

Finally, the EC of $U_{1}$ can be obtained as shown below

(37) $$\begin{array}{cc} {\bar{C}}_{1}= & E\left\{\frac{1}{2}\log_{2}\left(1+\underset{Y}{\underset{︸}{\max\left(\gamma_{U_{1}},\gamma_{RU_{1},x_{1}}\right)}}\right)\right\}\\ = & \frac{1}{2\ln2}\int_{0}^{\infty }\frac{1}{1+x}\left[1-F_{Y}\left(x\right)\right]dx. \end{array}$$

The CDF of *Y* is calculated as follows

(38) $$\begin{array}{c} F_{Y}\left(x\right)=\mathrm{Pr}\left(\max\left(\gamma_{U_{1}},\gamma_{RU_{1},x_{1}}\right)<x\right)\\ =1-e^{-\frac{\mu_{U_{1}}}{a_{1}\rho }x}\sum_{t=0}^{m_{U_{1}}-1}\frac{\mu_{U_{1}}^{t}x^{t}}{t!{\left(a_{1}\rho \right)}^{t}}\left[1-2\sum_{k=0}^{m_{R}N-1}\sum_{r=0}^{k}\sum_{d=0}^{m_{RU_{1}}N-1}\right.\\ \times \left(\begin{array}{c} k\\ r \end{array}\right)\left(\begin{array}{c} m_{RU_{1}}N-1\\ d \end{array}\right)\frac{\mu_{RU_{1}}^{m_{RU_{1}}N}\mu_{R}^{k}e^{-\frac{x}{a_{1}\rho }\left(\mu_{RU_{1}}+\mu_{R}\right)}}{k!\Gamma \left(m_{RU_{1}}N\right)}\\ \times {\left(\frac{x}{a_{1}\rho }+\rho^{-1}\right)}^{r}\left(1-e^{-\frac{\mu_{U_{1}}}{a_{1}\rho }x}\sum_{t=0}^{m_{U_{1}}-1}\frac{\mu_{U_{1}}^{t}x^{t}}{t!{\left(a_{1}\rho \right)}^{t}}\right)\\ \times {\left(\frac{\mu_{R}}{\mu_{RU_{1}}a_{1}\rho }\left(\frac{x}{a_{1}\rho }+\rho^{-1}\right)x\right)}^{\frac{d-r+1}{2}}{\left(\frac{x}{a_{1}\rho }\right)}^{m_{RU_{1}}N+k-d-1}\\ \times \left.K_{d-r+1}\left(2\sqrt{\frac{\mu_{R}\mu_{RU_{1}}}{a_{1}\rho }\left(\frac{x}{a_{1}\rho }+\rho^{-1}\right)x}\right)\right]. \end{array}$$

By replacing (38) in (37), $C_{1}$ is given by

(39) $${\bar{C}}_{1}=\frac{1}{2\ln2}\left({\bar{E}}_{1}+{\bar{E}}_{2}\right).$$

where

(40) $${\bar{E}}_{1}=\sum_{t=0}^{m_{U_{1}}-1}\frac{\mu_{U_{1}}^{t}}{t!{\left(a_{1}\rho \right)}^{t}}\int_{0}^{\infty }\frac{x^{t}e^{-\frac{\mu_{U_{1}}}{a_{1}\rho }x}}{1+x}dx.$$

Similar to solving (A17) and (A18), this can be achieved ${\bar{E}}_{1}$ as

(41) $${\bar{E}}_{1}=\sum_{t=0}^{m_{U_{1}}-1}\frac{\mu_{U_{1}}^{t}}{t!{\left(a_{1}\rho \right)}^{t}}G_{1,2}^{2,1}\left(\frac{\mu_{U_{1}}}{a_{1}\rho }x\left|\begin{array}{c} 1-t-1,-\\ 1-t-1,0 \end{array}\right.\right).$$

Next, we have ${\bar{E}}_{2}$, which is calculated as

(42) $$\begin{array}{cc} \; & {\bar{E}}_{2}=2\sum_{k=0}^{m_{R}N-1}\sum_{r=0}^{k}\sum_{d=0}^{m_{RU_{1}}N-1}\left(\begin{array}{c} k\\ r \end{array}\right)\left(\begin{array}{c} m_{RU_{1}}N-1\\ d \end{array}\right)\\ \; & \times \frac{\mu_{RU_{1}}^{m_{RU_{1}}N}\mu_{R}^{k}}{k!\Gamma \left(m_{RU_{1}}N\right)}\int_{0}^{\infty }\frac{1}{1+x}\left(1-e^{-\frac{\mu_{U_{1}}}{a_{1}\rho }x}\sum_{t=0}^{m_{U_{1}}-1}\frac{\mu_{U_{1}}^{t}x^{t}}{t!{\left(a_{1}\rho \right)}^{t}}\right)\\ \; & \times {\left(\frac{x}{a_{1}\rho }+\rho^{-1}\right)}^{r}{\left(\frac{x}{a_{1}\rho }\right)}^{m_{RU_{1}}N+k-d-1}\\ \; & \times {\left(\frac{\mu_{R}}{\mu_{RU_{1}}}\left(\frac{x}{a_{1}\rho }+\rho^{-1}\right)x\right)}^{\frac{d-r+1}{2}}e^{-\frac{x}{a_{1}\rho }\left(\mu_{RU_{1}}+\mu_{R}\right)}\\ \; & \times K_{d-r+1}\left(2\sqrt{\mu_{R}\mu_{RU_{1}}\left(\frac{x}{a_{1}\rho }+\rho^{-1}\right)x}\right). \end{array}$$

Similar to solving (A17) and (A18), this can be achieved ${\bar{E}}_{2}$ as

(43) $$\begin{array}{cc} {\bar{E}}_{2}\approx & \frac{\pi^{2}}{2J}\sum_{k=0}^{m_{R}N-1}\sum_{r=0}^{k}\sum_{d=0}^{m_{RU_{1}}N-1}\sum_{j=1}^{J}\left(\begin{array}{c} k\\ r \end{array}\right)\left(\begin{array}{c} m_{RU_{1}}N-1\\ d \end{array}\right)\\ \; & \times \frac{\sqrt{1-\varphi_{j}^{2}}}{1+\Lambda \left(\varphi_{j}\right)}\left(1-e^{-\mu_{U_{1}}\Theta \left(\varphi_{j}\right)}\sum_{t=0}^{m_{U_{1}}-1}\frac{\mu_{U_{1}}^{t}\Theta {\left(\varphi_{j}\right)}^{t}}{t!}\right)\\ \; & \times \frac{\mu_{RU_{1}}^{m_{RU_{1}}N}\mu_{R}^{k}}{k!\Gamma \left(m_{RU_{1}}N\right)}\sec^{2}\left(\frac{\pi }{4}\left(\Lambda \left(\varphi_{j}\right)+1\right)\right){\left(\Theta \left(\varphi_{j}\right)+\rho^{-1}\right)}^{r}\\ \; & \times e^{-\Theta \left(\varphi_{j}\right)\left(\mu_{RU_{1}}+\mu_{R}\right)}\Theta {\left(\varphi_{j}\right)}^{m_{RU_{1}}N+k-d-1}\\ \; & \times {\left(\frac{\mu_{R}\Xi \left(r\right)}{\mu_{RU_{1}}}\right)}^{\frac{d-r+1}{2}}K_{d-r+1}\left(2\sqrt{\mu_{R}\mu_{RU_{1}}\Xi \left(r\right)}\right), \end{array}$$

where $\varphi_{j}=\cos\left(\frac{2j-1}{2J}\pi \right)$, $\Lambda \left(r\right)=\tan\left(\frac{\pi \left(r+1\right)}{4}\right)$, $\Theta \left(r\right)=\frac{\Lambda \left(r\right)}{a_{1}\rho }$ and $\Xi \left(r\right)=\Theta \left(r\right)\left(\Theta \left(r\right)+\rho^{-1}\right)$.

As such, the EC of $U_{1}$ is given in (39).

## 6. Figure 2: Ergodic Capacity Analysis

Ergodic Rate of $U_{2}$: On the condition that $U_{2}$ can detect $x_{2}$, the achievable rate of $U_{2}$ can be written as $C_{2}^{II}=E\left\{\frac{1}{2}\log_{2}\left(1+{\bar{\gamma }}_{RU_{2},x_{2}}^{n^{*}}\right)\right\}$. The EC of $U_{2}$ can be obtained in the following proposition.

**Proposition** **5.** *The closed-form expression of approximated $C_{2}^{II}$ for $U_{2}$ is given by*

(44) $$\begin{array}{cc} C_{2}^{II}\approx & \frac{a_{2}\pi }{4a_{1}Q\ln2}\sum_{k=1}^{K}\frac{\sqrt{1-\phi_{k}^{2}}}{1+\Lambda \left(\phi_{k}\right)}\langle \frac{\mu_{R}^{m_{R}N}e^{-\mu_{R}{\tilde{\theta }}_{2}\left(\phi_{k}\right)\rho }}{\Gamma \left(m_{R}N\right)}\sum_{q=0}^{m_{R}N-1}\left(\begin{array}{c} m_{R}N-1\\ q \end{array}\right)\\ \; & \times {\left({\tilde{\theta }}_{2}\left(\phi_{k}\right)\rho \right)}^{m_{R}N-q-1}\left[\frac{q!}{\mu_{R}^{q+1}}-2\sum_{a=0}^{N}\left(\begin{array}{c} N\\ a \end{array}\right){\left(-1\right)}^{a}e^{-\mu_{RU_{2}}a{\tilde{\theta }}_{2}\left(\phi_{k}\right)\rho }\right.\\ \; & \times \sum_{j_{0}+\ldots +j_{m_{RU_{2}}-1}=a}\left(\begin{array}{c} a\\ j_{0},\ldots ,j_{m_{RU_{2}}-1} \end{array}\right)\prod_{b=0}^{m_{RU_{2}}-1}{\left(\frac{\mu_{RU_{2}}^{b}}{b!}\right)}^{j_{b}}\sum_{c=0}^{bj_{b}}\left(\begin{array}{c} bj_{b}\\ c \end{array}\right)\\ \; & \times {\left[{\tilde{\theta }}_{2}\left(\phi_{k}\right)\left({\tilde{\theta }}_{2}\left(\phi_{k}\right)\rho^{2}+1\right)\right]}^{c}{\left({\tilde{\theta }}_{2}\left(\phi_{k}\right)\rho \right)}^{bj_{b}-c}\\ \; & \times {\left(\frac{\mu_{RU_{2}}a{\tilde{\theta }}_{2}\left(\phi_{k}\right)\left({\tilde{\theta }}_{2}\left(\phi_{k}\right)\rho^{2}+1\right)}{\mu_{R}}\right)}^{\frac{q-c+1}{2}}\\ \; & \left.\times K_{q-c+1}\left(2\sqrt{\mu_{R}\mu_{RU_{2}}a{\tilde{\theta }}_{2}\left(\phi_{k}\right)\left({\tilde{\theta }}_{2}\left(\phi_{k}\right)\rho^{2}+1\right)}\right)\right]\rangle , \end{array}$$

*where $\phi_{k}=\cos\left(\frac{2k-1}{2K}\pi \right)$.*

**Proof.**  From (34) into $C_{2}^{II}$, the EC of $U_{2}$ is written as

(45) $$\begin{array}{cc} C_{2}^{II}= & \frac{1}{2\ln2}\int_{0}^{\frac{a_{2}}{a_{1}}}\frac{1}{1+x}\left[1-F_{X_{1}}\left(\frac{x}{a_{2}-xa_{1}}\right)\right]dx\\ = & \frac{1}{2\ln2}\int_{0}^{\frac{a_{2}}{a_{1}}}\frac{1}{1+x}\langle \frac{\mu_{R}^{m_{R}N}e^{-\mu_{R}\theta_{2}\left(x\right)\rho }}{\Gamma \left(m_{R}N\right)}\sum_{q=0}^{m_{R}N-1}\left(\begin{array}{c} m_{R}N-1\\ q \end{array}\right)\\ \; & \times {\left(\theta_{2}\left(x\right)\rho \right)}^{m_{R}N-q-1}\left[\frac{q!}{\mu_{R}^{q+1}}-2\sum_{a=0}^{N}\left(\begin{array}{c} N\\ a \end{array}\right){\left(-1\right)}^{a}\right.\\ \; & \times e^{-\mu_{RU_{2}}a\theta_{2}\left(x\right)\rho }\sum_{j_{0}+\ldots +j_{m_{RU_{2}}-1}=a}\left(\begin{array}{c} a\\ j_{0},\ldots ,j_{m_{RU_{2}}-1} \end{array}\right)\\ \; & \times \prod_{b=0}^{m_{RU_{2}}-1}{\left(\frac{\mu_{RU_{2}}^{b}}{b!}\right)}^{j_{b}}\sum_{c=0}^{bj_{b}}\left(\begin{array}{c} bj_{b}\\ c \end{array}\right){\left[\theta_{2}\left(x\right)\left(\theta_{2}\left(x\right)\rho^{2}+1\right)\right]}^{c}\\ \; & \times {\left(\theta_{2}\left(x\right)\rho \right)}^{bj_{b}-c}{\left(\frac{\mu_{RU_{2}}a\theta_{2}\left(x\right)\left(\theta_{2}\left(x\right)\rho^{2}+1\right)}{\mu_{R}}\right)}^{\frac{q-c+1}{2}}\\ \; & \left.\times K_{q-c+1}\left(2\sqrt{\mu_{R}\mu_{RU_{2}}a\theta_{2}\left(x\right)\left(\theta_{2}\left(x\right)\rho^{2}+1\right)}\right)\right]\rangle dx, \end{array}$$

where $X_{1}=\frac{a_{2}\rho^{2}Y{\bar{Z}}_{2}^{n^{*}}}{a_{1}\rho^{2}Y{\bar{Z}}_{2}^{n^{*}}+\rho Y+\rho {\bar{Z}}_{2}^{n^{*}}+1}$ and $\theta_{2}\left(x\right)=\frac{x}{\rho^{2}\left(a_{2}-xa_{1}\right)}$.

Let $t=\frac{2a_{1}}{a_{2}}x-1\Rightarrow \frac{a_{2}\left(t+1\right)}{2a_{1}}=x\Rightarrow \frac{a_{2}}{2a_{1}}dt=dx$; then, $C_{2}^{II}$ is calculated as

(46) $$\begin{array}{cc} C_{2}^{II}= & \frac{a_{2}}{4a_{1}\ln2}\int_{-1}^{1}\frac{1}{1+\Lambda \left(t\right)}\langle \frac{\mu_{R}^{m_{R}N}e^{-\mu_{R}{\tilde{\theta }}_{2}\left(t\right)\rho }}{\Gamma \left(m_{R}N\right)}\sum_{q=0}^{m_{R}N-1}\left(\begin{array}{c} m_{R}N-1\\ q \end{array}\right){\left({\tilde{\theta }}_{2}\left(t\right)\rho \right)}^{m_{R}N-q-1}\\ \; & \times \left[\frac{q!}{\mu_{R}^{q+1}}-2\sum_{a=0}^{N}\left(\begin{array}{c} N\\ a \end{array}\right){\left(-1\right)}^{a}e^{-\mu_{RU_{2}}a{\tilde{\theta }}_{2}\left(t\right)\rho }\sum_{j_{0}+\ldots +j_{m_{RU_{2}}-1}=a}\left(\begin{array}{c} a\\ j_{0},\ldots ,j_{m_{RU_{2}}-1} \end{array}\right)\right.\\ \; & \times \prod_{b=0}^{m_{RU_{2}}-1}{\left(\frac{\mu_{RU_{2}}^{b}}{b!}\right)}^{j_{b}}\sum_{c=0}^{bj_{b}}\left(\begin{array}{c} bj_{b}\\ c \end{array}\right){\left[{\tilde{\theta }}_{2}\left(t\right)\left({\tilde{\theta }}_{2}\left(t\right)\rho^{2}+1\right)\right]}^{c}\\ \; & \times {\left({\tilde{\theta }}_{2}\left(t\right)\rho \right)}^{bj_{b}-c}{\left(\frac{\mu_{RU_{2}}a{\tilde{\theta }}_{2}\left(t\right)\left({\tilde{\theta }}_{2}\left(t\right)\rho^{2}+1\right)}{\mu_{R}}\right)}^{\frac{q-c+1}{2}}\\ \; & \left.\times K_{q-c+1}\left(2\sqrt{\mu_{R}\mu_{RU_{2}}a{\tilde{\theta }}_{2}\left(t\right)\left({\tilde{\theta }}_{2}\left(t\right)\rho^{2}+1\right)}\right)\right]\rangle dt, \end{array}$$

where $\Lambda \left(t\right)=\frac{a_{2}\left(t+1\right)}{2a_{1}}$ and ${\tilde{\theta }}_{2}\left(t\right)=\frac{\Lambda \left(t\right)}{\rho^{2}\left(a_{2}-\Lambda \left(t\right)a_{1}\right)}$.

Although obtaining a closed-form formula for $C_{2}^{II}$ is challenging, we can acquire an accurate approximation for it. We may obtain (44) by using Gaussian–Chebyshev quadrature Equation (25.4.38) [49].

The proof is finished. □

Ergodic Rate of $U_{1}$: If $U_{1}$ is capable of detecting $x_{1}$, the EC of $U_{1}$ may be calculated as

(47) $$\begin{array}{cc} C_{1}^{II}= & E\left\{\frac{1}{2}\log_{2}\left(1+{\bar{\gamma }}_{RU_{1},x_{1}}^{n^{*}}\right)\right\}\\ = & \frac{1}{2\ln2}\int_{0}^{\infty }\frac{1-F_{X_{2}}\left(x\right)}{1+x}dx\\ = & \frac{1}{2\ln2}\int_{0}^{\infty }\frac{1}{1+x}\langle \frac{\mu_{R}^{m_{R}N}e^{-\mu_{R}\theta_{1}\left(x\right)\rho }}{\Gamma \left(m_{R}N\right)}\sum_{q=0}^{m_{R}N-1}\left(\begin{array}{c} m_{R}N-1\\ q \end{array}\right){\left(\theta_{1}\left(x\right)\rho \right)}^{m_{R}N-q-1}\\ \; & \times \left[\frac{q!}{\mu_{R}^{q+1}}-\sum_{a=0}^{N}\left(\begin{array}{c} N\\ a \end{array}\right){\left(-1\right)}^{a}\right.e^{-\mu_{RU_{1}}a\theta_{1}\left(x\right)\rho }\sum_{j_{0}+\ldots +j_{m_{RU_{1}}-1}=a}\left(\begin{array}{c} a\\ j_{0},\ldots ,j_{m_{RU_{1}}-1} \end{array}\right)\\ \; & \times \prod_{b=0}^{m_{RU_{1}}-1}{\left(\frac{\mu_{RU_{1}}^{b}}{b!}\right)}^{j_{b}}\sum_{c=0}^{bj_{b}}\left(\begin{array}{c} bj_{b}\\ c \end{array}\right){\left[\theta_{1}\left(x\right)\left(\theta_{1}\left(x\right)\rho^{2}+1\right)\right]}^{c}\\ \; & \times {\left(\theta_{1}\left(x\right)\rho \right)}^{bj_{b}-c}2{\left(\frac{\mu_{RU_{1}}a\theta_{1}\left(x\right)\left(\theta_{1}\left(x\right)\rho^{2}+1\right)}{\mu_{R}}\right)}^{\frac{q-c+1}{2}}\\ \; & \left.\times K_{q-c+1}\left(2\sqrt{\mu_{R}\mu_{RU_{1}}a\theta_{1}\left(x\right)\left(\theta_{1}\left(x\right)\rho^{2}+1\right)}\right)\right]\rangle dx, \end{array}$$

where $X_{2}=\frac{a_{1}\rho^{2}Y{\bar{Z}}_{1}^{n^{*}}}{\rho Y+\rho {\bar{Z}}_{1}^{n^{*}}+1}$ and $\theta_{1}\left(x\right)=\frac{x}{a_{1}\rho^{2}}$.

After some steps, the EC of $U_{1}$ in Figure 2 can be obtained as (48), where step (a) follows by letting $t=\frac{4}{\pi }\arctan\left(x\right)-1$ and step (b) follows by using Gaussian–Chebyshev quadrature approximation [49], in which $\Xi \left(t\right)=\tan\left(\frac{\pi \left(t+1\right)}{4}\right)$, ${\tilde{\theta }}_{1}\left(t\right)=\frac{\Xi \left(t\right)}{a_{1}\rho^{2}}$, sec2x=11cos2xcos2x, *K* is a parameter that determines the trade-off between complexity and accuracy $\phi_{k}=\cos\left(\frac{2k-1}{2K}\pi \right)$.

(48) $$\begin{array}{cc} C_{1}^{II}\overset{\left(a\right)}{=} & \frac{\pi }{8\ln2}\int_{1}^{-1}\frac{\sec^{2}\left(\frac{\pi }{4}\left(t+1\right)\right)}{\left[1+\Xi \left(t\right)\right]}\langle \frac{\mu_{R}^{m_{R}N}e^{-\mu_{R}{\tilde{\theta }}_{1}\left(t\right)\rho }}{\Gamma \left(m_{R}N\right)}\sum_{q=0}^{m_{R}N-1}\left(\begin{array}{c} m_{R}N-1\\ q \end{array}\right){\left({\tilde{\theta }}_{1}\left(t\right)\rho \right)}^{m_{R}N-q-1}\\ \; & \times \left[\frac{q!}{\mu_{R}^{q+1}}-2\sum_{a=0}^{N}\left(\begin{array}{c} N\\ a \end{array}\right){\left(-1\right)}^{a}\right.e^{-\mu_{RU_{1}}a{\tilde{\theta }}_{1}\left(t\right)\rho }\sum_{j_{0}+\ldots +j_{m_{RU_{1}}-1}=a}\left(\begin{array}{c} a\\ j_{0},\ldots ,j_{m_{RU_{1}}-1} \end{array}\right)\\ \; & \times \prod_{b=0}^{m_{RU_{1}}-1}{\left(\frac{\mu_{RU_{1}}^{b}}{b!}\right)}^{j_{b}}\sum_{c=0}^{bj_{b}}\left(\begin{array}{c} bj_{b}\\ c \end{array}\right){\left[{\tilde{\theta }}_{1}\left(t\right)\left({\tilde{\theta }}_{1}\left(t\right)\rho^{2}+1\right)\right]}^{c}\\ \; & \times {\left({\tilde{\theta }}_{1}\left(t\right)\rho \right)}^{bj_{b}-c}{\left(\frac{\mu_{RU_{1}}a{\tilde{\theta }}_{1}\left(t\right)\left({\tilde{\theta }}_{1}\left(t\right)\rho^{2}+1\right)}{\mu_{R}}\right)}^{\frac{q-c+1}{2}}\\ \; & \left.\times K_{q-c+1}\left(2\sqrt{\mu_{R}\mu_{RU_{1}}a{\tilde{\theta }}_{1}\left(t\right)\left({\tilde{\theta }}_{1}\left(t\right)\rho^{2}+1\right)}\right)\right]\rangle dt\\ \overset{\left(b\right)}{\approx } & \frac{\pi^{2}}{8K\ln2}\sum_{k=1}^{K}\frac{\sqrt{1-\phi_{k}^{2}}}{\left[1+\Xi \left(t\right)\right]}\sec^{2}\left(\frac{\pi }{4}\left(t+1\right)\right)\langle \frac{\mu_{R}^{m_{R}N}e^{-\mu_{R}{\tilde{\theta }}_{1}\left(\phi_{k}\right)\rho }}{\Gamma \left(m_{R}N\right)}\\ \; & \times \sum_{q=0}^{m_{R}N-1}\left(\begin{array}{c} m_{R}N-1\\ q \end{array}\right){\left({\tilde{\theta }}_{1}\left(\phi_{k}\right)\rho \right)}^{m_{R}N-q-1}\left[\frac{q!}{\mu_{R}^{q+1}}-2\sum_{a=0}^{N}\left(\begin{array}{c} N\\ a \end{array}\right){\left(-1\right)}^{a}\right.\\ \; & \times e^{-\mu_{RU_{1}}a{\tilde{\theta }}_{1}\left(\phi_{k}\right)\rho }\sum_{j_{0}+\ldots +j_{m_{RU_{1}}-1}=a}\left(\begin{array}{c} a\\ j_{0},\ldots ,j_{m_{RU_{1}}-1} \end{array}\right)\prod_{b=0}^{m_{RU_{1}}-1}{\left(\frac{\mu_{RU_{1}}^{b}}{b!}\right)}^{j_{b}}\\ \; & \times \sum_{c=0}^{bj_{b}}\left(\begin{array}{c} bj_{b}\\ c \end{array}\right){\left[{\tilde{\theta }}_{1}\left(\phi_{k}\right)\left({\tilde{\theta }}_{1}\left(\phi_{k}\right)\rho^{2}+1\right)\right]}^{c}{\left({\tilde{\theta }}_{1}\left(\phi_{k}\right)\rho \right)}^{bj_{b}-c}\\ \; & \times {\left(\frac{\mu_{RU_{1}}a{\tilde{\theta }}_{1}\left(\phi_{k}\right)\left({\tilde{\theta }}_{1}\left(\phi_{k}\right)\rho^{2}+1\right)}{\mu_{R}}\right)}^{\frac{q-c+1}{2}}\\ \; & \left.\times K_{q-c+1}\left(2\sqrt{\mu_{R}\mu_{RU_{1}}a{\tilde{\theta }}_{1}\left(\phi_{k}\right)\left({\tilde{\theta }}_{1}\left(\phi_{k}\right)\rho^{2}+1\right)}\right)\right]\rangle . \end{array}$$

## 7. Simulation Results

In this section, we set $m=m_{U_{1}}=m_{U_{2}}=m_{R}=m_{RU_{1}}=m_{RU_{2}}$ and numerically simulate some theoretical results to show the outage performance. In particular, the main parameters can be seen in Table 2. In addition, the Gaussian-Chebyshev parameter is selected as D=J=K=100 to yield a close approximation.

**Figure 4:**
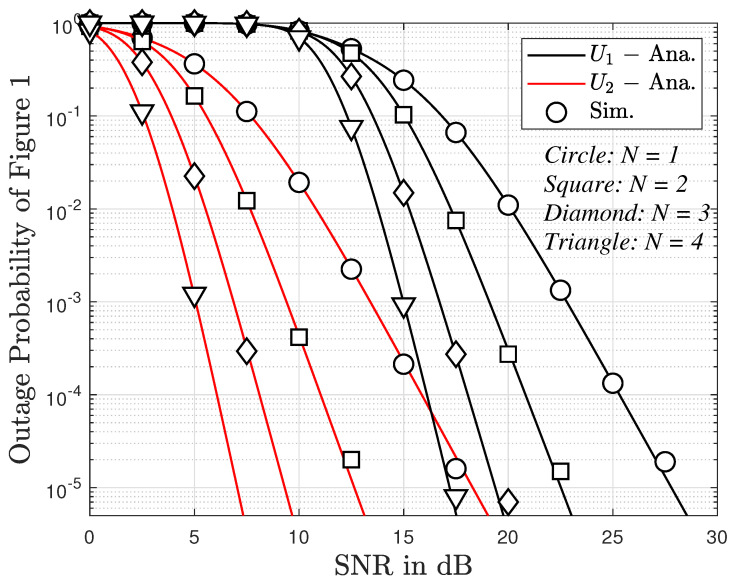
Outage probability versus transmitting SNR of two users with direct link.

In Figure 4, we examined the outage probability versus SNR and the number of antennas. The figure shows that the outage probability for User 2 is lower than that for user 1. Although the outage probability for both users tends to decrease linearly and quickly, the outage probability for User 2 still keeps a value of around $10^{0}$ in the SNR range from 0 to 10 dB. In particular, when the number of antennas increases from 1 to 3, the outage probability for both users is reduced. This implies that the more antennas, the more reliable the system.

**Figure 5:**
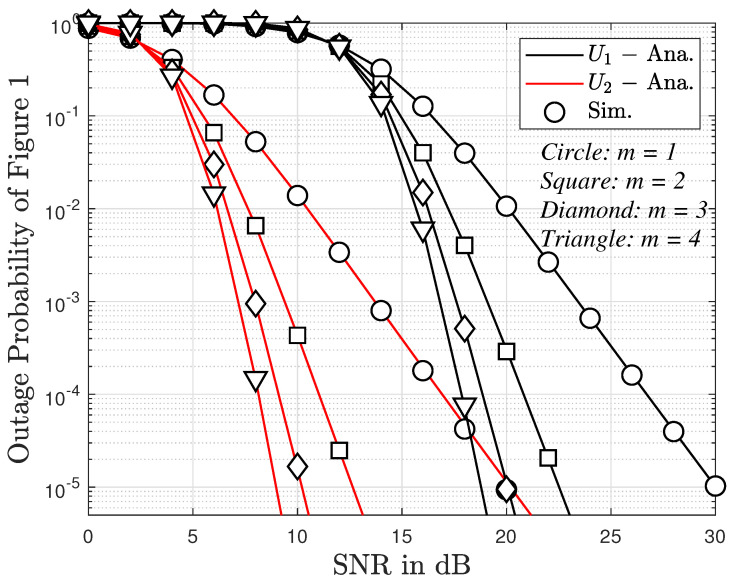
The outage probability versus SNR and different values of *m*, with N=2 with direct link.

In Figure 5, we present the outage probability versus SNR and fading severity factor *m*. Similar to Figure 4, the outage probability for User 2 is also lower than that for User 1. The outage probability for both users decreases quickly as SNR increases. For impacts of *m* on the outage probability, it is observed from the figure that the larger the value of *m*, the higher the slope of the outage probability curves. This can be explained based on (15) and (17).

**Figure 6:**
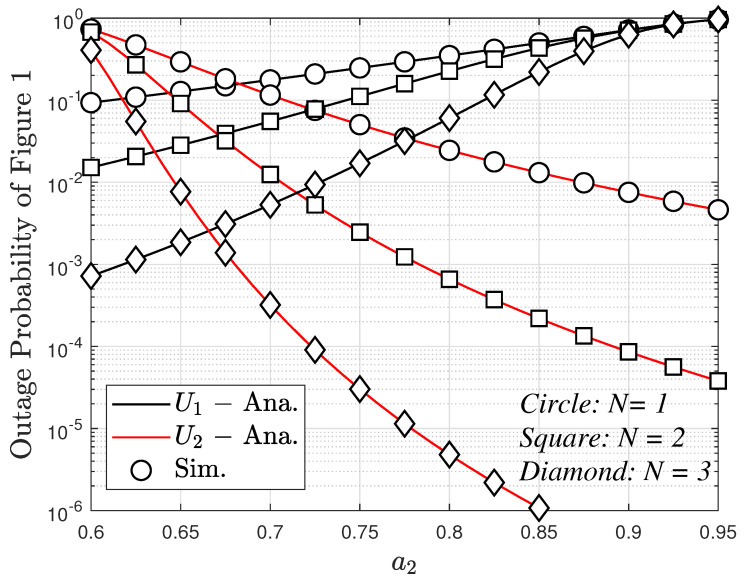
The outage probability versus $a_{2}$ with ρ=10 (dB), m=2 and different values of *N* with direct link.

Figure 6 plots the outage probability versus power allocation coefficient $a_{2}$. The figure shows that the outage probability for User 2 decreases in the case of increasing $a_{2}$. However, this is the opposite for User 1 where the outage probability increases linearly when $a_{2}$ increases. In addition, the outage probability for two users tends to decrease since the number of antennas increases.

**Figure 7:**
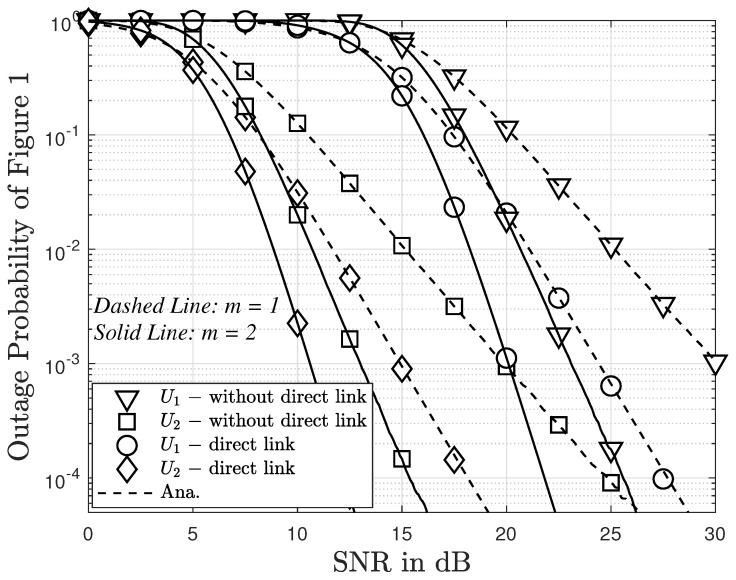
The outage probability with/without direct link versus $\varepsilon_{th}=3$ (dB) and N=2.

In Figure 7, we present the relationship between the outage probability and SNR for scenarios with and without direct links between BS and users for Figure 1. It is evident that the outage probability for User 2 consistently remains lower than that for User 1. Furthermore, when there is no direct link between BS and users, the outage probability for User 1 is higher compared to User 2. The absence of a direct link leads to a higher outage probability compared to scenarios with a direct link. Interestingly, as the value of *m* increases from 1 to 2, the outage probability for both users, in cases with or without direct links, consistently improves.

**Figure 8:**
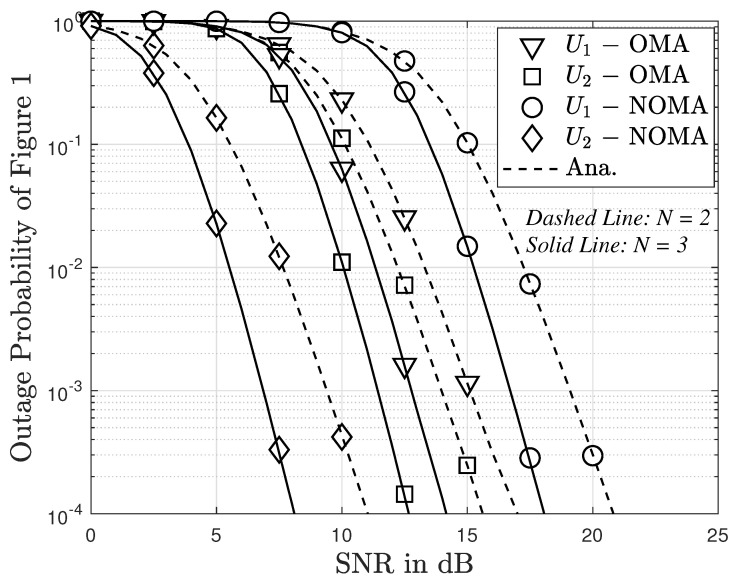
Comparison of outage probability between OMA and NOMA versus SNR with m=2 with direct link.

Figure 8 presents the outage probability as a function of SNR in dB in cases of NOMA and OMA schemes. It is observed from the figure that the outage probability for User 2 and NOMA is lower than that for OMA in both cases where the number of antennas is 1 and 2. However, this is the opposite for User 1 where the outage probability for OMA is lower than that for NOMA. In general, the outage probability curves decrease quickly as SNR increases.

**Figure 9:**
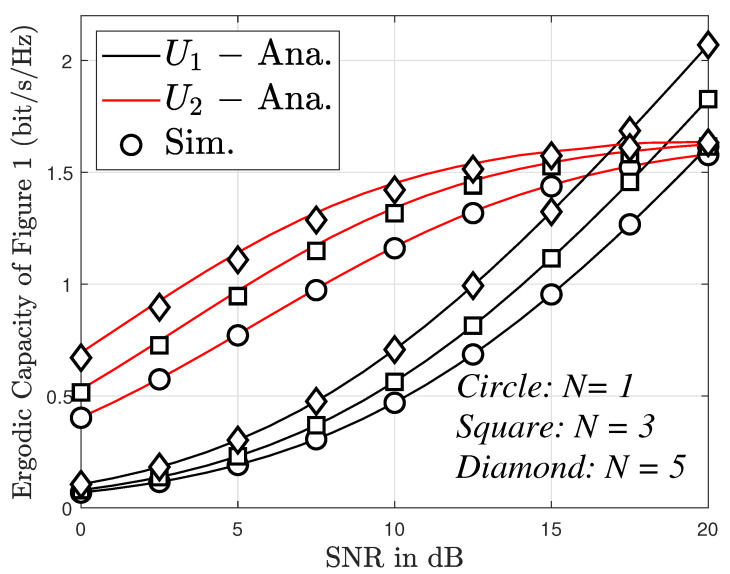
The ergodic capacity versus SNR and different values of *N*, with m=2 with direct link.

In Figure 9, the ergodic capacity versus SNR in different cases of antennas (i.e., *n* = 1, 3, 5) was investigated. The figure shows that the ergodic capacity of User 2 increases gradually in the SNR range of 0 to 20 dB. However, this tends to remain constant during the SNR range of 20 to 30 dB. For User 1, the ergodic capacity increases rapidly as SNR varies from 10 to 30 dB. In addition, the number of antennas also causes a change in the ergodic capacity. Specifically, the higher the number of antennas, the larger the ergodic capacity. However, the impact of the number of antennas on the ergodic capacity for User 2 is insignificant, particularly in the SNR range of 20 to 30 dB.

**Figure 10:**
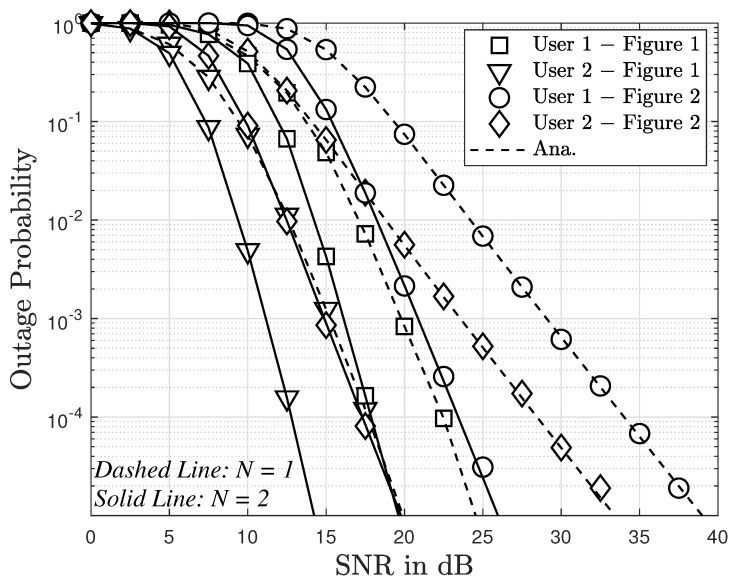
Outage comparison between Figure 1 and Figure 2, with m=2.

Next, Figure 10 demonstrates that the performance of two users in Figure 1 is better than that of the users Figure 2 in terms of outage probability. The main reason is that Figure 1 still exhibits higher diversity, although Figure 1 requires a higher design cost. The demand for low-cost design for some applications of the Internet of Things would prefer the benefits of Figure 2.

**Figure 11:**
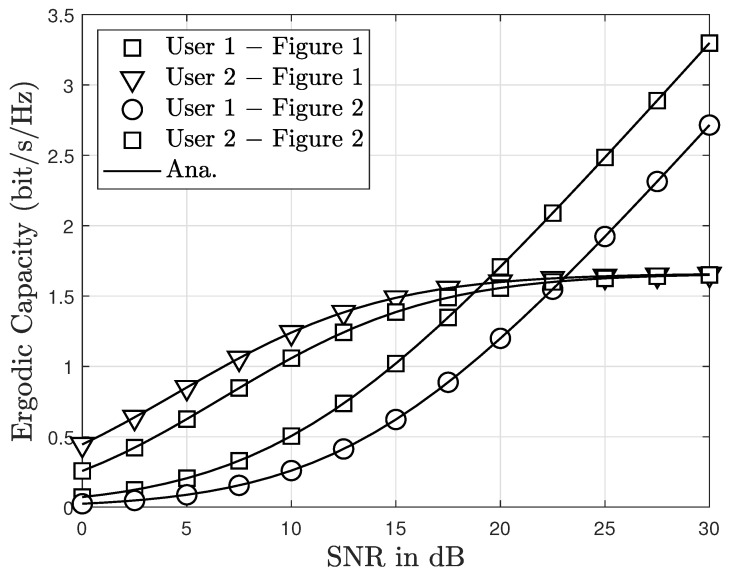
Ergodic capacity between Figure 1 and Figure 2 with m=2 and N=2.

Figure 11 presents the inclusion of simulation data for comparative analysis, serving to verify the accuracy of the obtained analytical results. Additionally, a comparison is made between the ergodic capabilities of the proposed system in Figure 1 and Figure 2. Specifically, User 2 demonstrates a higher ergodic capacity in Figure 1 compared to Figure 2 within the low and moderate SNR regions. However, User 1 achieves a superior ergodic capacity over Figure 2 across a wide range of SNR values. Remarkably, in the medium and high SNR ranges, User 2’s achievable capacity converges to a constant because interferences in the instantaneous SINRs at User 2 rise as the average SNR increases. Conversely, the interferences experienced by User 1 intensify as the average SNR rises, as demonstrated in Equations (36), (39), (44) and (48). Consequently, it is evident that the ergodic capacity of User 2 in Figure 1 outperforms that of User 2 in Figure 2.

**Figure 12:**
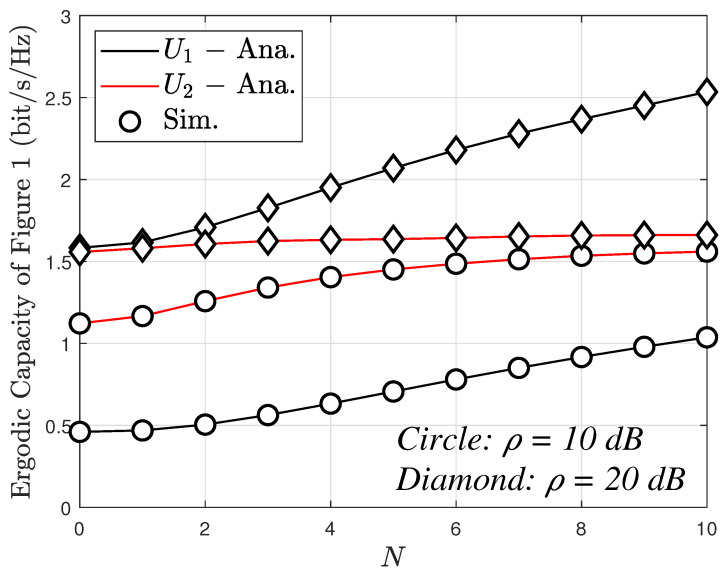
The ergodic capacity versus *N* with m=2 with direct link.

Lastly, Figure 12 illustrates the variation of the ergodic capacity as the number of antennas increases, ranging up to 10. Notably, User 1 exhibits a higher ergodic capacity compared to User 2. The ergodic capacity demonstrates a proportional relationship with the number of antennas. However, for User 2, this capacity remains nearly constant when the number of antennas exceeds 4. This observation suggests that the impact of the number of antennas on User 1 is greater than that on User 2. This can be explained based on (36) and (39).

## 8. Conclusions and Future Work

This paper has presented two practical schemes of multiple-antennas UAV-aided IoT systems. Closed-form expressions for the outage probability were studied to confirm Figure 1 with a higher superiority of outage performance compared with Figure 2. We also provide performance analysis for the system by deriving a closed-form approximation of the ergodic capacity in Figure 1. The numerical results show that a weak user (User 2) achieves a lower probability than a strong user (User 1). The outage probability without direct links is lower than that with direct links. The IoT relying on the NOMA scheme is superior to that using the OMA scheme. The performance of the system increases when the number of antennas increases. These findings are a basic background for investigating the performance of the NOMA system with multiple antennas. In future work, reconfigurable intelligent surface-based UAVs could be studied to improve the performance of IoT users. In the future, we will combine UAV NOMA with changeable intelligent surfaces to increase system performance metrics even more.

## Figures and Tables

**Table 1:** Comparison of the proposed scheme with similar ideas.

	Our Scheme	[1]	[34]	[35]	[36]	[37]	[38]
NOMA	X	X	X				
Multiple-Antennas UAV	X						
Multiple UAV		X					
Maximum Ratio Transmission	X						
Transmit Antenna Selection	X						
Amplify and Forward	X						
Interference		X	X			X	
Outage Probability	X						
Ergodic Capacity	X						
Nakagami-*m* Channel	X						
Optimization		X	X	X	X		X

**Table 2:** Definition of system parameters.

Parameters	Notation	Values
The power allocation coefficient	$\left\{a_{1},a_{2}\right\}$	$\left\{0.1,0.9\right\}$
The fading severity parameter	*m*	2
Target rates SINR	$\varepsilon_{th}$	2 (dB)
Antennas at *R*	*N*	$\left\{1,2,3\right\}$
The average power	$\left\{\lambda_{U_{1}},\lambda_{U_{2}}\right\}$	$\left\{1,1\right\}$
	$\lambda_{R}$	1
	$\lambda_{RU_{1}}$	0.5
	$\lambda_{RU_{2}}$	0.9

## Appendix A

**Proof** **Proposition 1.** From (15), the outage probability at $U_{2}$ can be calculated by

(A1) $${\bar{P}}_{2}^{I}=\underset{{\bar{A}}_{1}}{\underset{︸}{\mathrm{Pr}\left(\frac{a_{2}\rho X_{2}}{a_{1}\rho X_{2}+1}<\varepsilon_{th}\right)}}\times \underset{{\bar{A}}_{2}}{\underset{︸}{1-\mathrm{Pr}\left(\gamma_{RU_{2},x_{2}}>\varepsilon_{th}\right)}}.$$

Applying (13a), ${\bar{A}}_{1}$ can be calculated as follows

(A2) $$\begin{array}{cc} {\bar{A}}_{1}= & \mathrm{Pr}\left(\frac{a_{2}\rho X_{2}}{a_{1}\rho X_{2}+1}<\varepsilon_{th}\right)\\ = & \mathrm{Pr}\left(X_{2}<\theta \right)\\ = & F_{X_{2}}\left(\theta \right)\\ = & 1-e^{-\mu_{U_{2}}\theta }\sum_{a=0}^{m_{U_{2}}-1}\frac{\mu_{U_{2}}^{a}\theta^{a}}{a!}, \end{array}$$

where $\theta =\frac{\varepsilon_{th}}{\rho \left(a_{2}-a_{1}\varepsilon_{th}\right)}$ Next, ${\bar{A}}_{2}$ can be computed as

(A3) $$\begin{array}{cc} {\bar{A}}_{2}= & 1-\mathrm{Pr}\left(Y_{n}Z_{2n}>\theta \left(Y_{n}+Z_{2n}+\rho^{-1}\right)\right)\\ = & 1-\mathrm{Pr}\left(Y_{n}>\frac{\theta \left(Z_{2n}+\rho^{-1}\right)}{Z_{2n}-\theta },Z_{2n}>\theta \right)\\ = & 1-\int_{\theta }^{\infty }f_{Z_{2n}}\left(x\right)\left[1-F_{Y_{n}}\left(\frac{\theta \left(x+\rho^{-1}\right)}{x-\theta }\right)\right]dx\\ = & 1-\sum_{b=0}^{m_{R}N-1}\frac{\mu_{RU_{2}}^{m_{RU_{2}}N}\mu_{R}^{b}}{b!\Gamma \left(m_{RU_{2}}N\right)}\int_{\theta }^{\infty }x^{m_{RU_{2}}N-1}e^{-\mu_{RU_{2}}x}\\ \; & \times e^{-\mu_{R}\frac{\theta \left(x+\rho^{-1}\right)}{x-\theta }}{\left[\frac{\theta \left(x+\rho^{-1}\right)}{x-\theta }\right]}^{b}dx. \end{array}$$

Let t=x−θ→t+θ=x→dt=dx; then, ${\bar{A}}_{2}$ is given by

(A4) $$\begin{array}{cc} {\bar{A}}_{2}= & 1-\sum_{b=0}^{m_{R}N-1}\frac{\mu_{RU_{2}}^{m_{RU_{2}}N}\mu_{R}^{b}e^{-\theta \left(\mu_{RU_{2}}+\mu_{R}\right)}}{b!\Gamma \left(m_{RU_{2}}N\right)}\int_{0}^{\infty }{\left(t+\theta \right)}^{m_{RU_{2}}N-1}\\ \; & \times e^{-\mu_{RU_{2}}t-\frac{\mu_{R}\theta \left(\theta +\rho^{-1}\right)}{t}}{\left[\theta +\frac{\theta^{2}+\theta \rho^{-1}}{t}\right]}^{b}dt. \end{array}$$

With the help of Equations (1.111), (3.471.9) [48] and after some manipulations, we have

(A5) $$\begin{array}{cc} {\bar{A}}_{2}= & 1-2\sum_{b=0}^{m_{R}N-1}\sum_{q=0}^{b}\sum_{n=0}^{m_{RU_{2}}N-1}\left(\begin{array}{c} b\\ q \end{array}\right)\left(\begin{array}{c} m_{RU_{2}}N-1\\ n \end{array}\right)\\ \; & \times \frac{\mu_{R}^{b}\theta^{m_{RU_{2}}N+b-q-n-1}e^{-\theta \left(\mu_{RU_{2}}+\mu_{R}\right)}}{b!\Gamma \left(m_{RU_{2}}N\right)}\\ \; & \times \mu_{RU_{2}}^{m_{RU_{2}}N}{\left(\theta^{2}+\theta \rho^{-1}\right)}^{q}{\left(\frac{\mu_{R}\theta \left(\theta +\rho^{-1}\right)}{\mu_{RU_{2}}}\right)}^{\frac{n-q+1}{2}}\\ \; & \times K_{n-q+1}\left(2\sqrt{\mu_{R}\mu_{RU_{2}}\theta \left(\theta +\rho^{-1}\right)}\right). \end{array}$$

Finally, by substituting (A5) and (A2) into (A1), a closed-form expression for the outage probability at $U_{2}$ can be derived as

(A6) $$\begin{array}{cc} {\bar{P}}_{2}^{I}= & \left(1-e^{-\mu_{U_{2}}\theta }\sum_{a=0}^{m_{U_{2}}-1}\frac{\mu_{U_{2}}^{a}\theta^{a}}{a!}\right)\left[1-2\sum_{b=0}^{m_{R}N-1}\sum_{q=0}^{b}\sum_{n=0}^{m_{RU_{2}}N-1}\right.\\ \; & \times 2\left(\begin{array}{c} b\\ q \end{array}\right)\left(\begin{array}{c} m_{RU_{2}}N-1\\ n \end{array}\right)\frac{{\left(\theta^{2}+\theta \rho^{-1}\right)}^{q}e^{-\theta \left(\mu_{RU_{2}}+\mu_{R}\right)}}{b!\Gamma \left(m_{RU_{2}}N\right)}\\ \; & \times \mu_{R}^{b}\theta^{m_{RU_{2}}N+b-q-n-1}{\left(\frac{\mu_{R}\left(\theta^{2}+\theta \rho^{-1}\right)}{\mu_{RU_{2}}}\right)}^{\frac{n-q+1}{2}}\\ \; & \left.\times \mu_{RU_{2}}^{m_{RU_{2}}N}K_{n-q+1}\left(2\sqrt{\mu_{R}\mu_{RU_{2}}\left(\theta^{2}+\theta \rho^{-1}\right)}\right)\right]. \end{array}$$

The proof is completed. □

## Appendix B

**Proof** **of Proposition 3.** The outage probability of $U_{2}$ with Figure 2 is calculated as

(A7) $$\begin{array}{c} {\bar{P}}_{1}^{II}=1-\mathrm{Pr}\left(Y{\bar{Z}}_{1}^{n^{*}}>\theta_{\max}\left(\rho Y+\rho {\bar{Z}}_{1}^{n^{*}}+1\right)\right)\\ =1-\mathrm{Pr}\left({\bar{Z}}_{1}^{n^{*}}>\frac{\theta_{\max}}{\left(Y-\theta_{\max}\rho \right)}\left(\rho Y+1\right)\right)\\ =1-\mathrm{Pr}\left({\bar{Z}}_{1}^{n^{*}}>\frac{\theta_{\max}}{\left(Y-\theta_{\max}\rho \right)}\left(\rho Y+1\right),Y>\theta_{\max}\rho \right)\\ =1-\int_{\theta_{\max}\rho }^{\infty }f_{Y}\left(x\right)\left[1-F_{{\bar{Z}}_{1}^{n^{*}}}\left[\frac{\theta_{\max}}{\left(x-\theta_{\max}\rho \right)}\left(\rho x+1\right)\right]\right]dx\\ =1-\frac{\mu_{R}^{m_{R}N}}{\Gamma \left(m_{R}N\right)}\int_{\theta_{\max}\rho }^{\infty }x^{m_{R}N-1}e^{-\mu_{R}x}\\ \times \left[1-\sum_{a=0}^{N}\left(\begin{array}{c} N\\ a \end{array}\right){\left(-1\right)}^{a}e^{-\mu_{RU_{1}}a\left[\frac{\theta_{\max}}{\left(x-\theta_{\max}\rho \right)}\left(\rho x+1\right)\right]}\right.\\ \times \sum_{j_{0}+\ldots +j_{m_{RU_{1}}-1}=a}\left(\begin{array}{c} a\\ j_{0},\ldots ,j_{m_{RU_{1}}-1} \end{array}\right)\prod_{b=0}^{m_{RU_{1}}-1}\\ \left.\times {\left(\frac{\mu_{RU_{1}}^{b}}{b!}\right)}^{j_{b}}{\left(\frac{\theta_{\max}}{\left(x-\theta_{\max}\rho \right)}\left(\rho x+1\right)\right)}^{bj_{b}}\right]dx. \end{array}$$

Let $t=x-\theta_{\max}\rho \to t+\theta_{\max}\rho =x\to dt=dx$; then, ${\bar{P}}_{1}^{II}$ is given by

(A8) $$\begin{array}{c} {\bar{P}}_{1}^{II}=1-\frac{\mu_{R}^{m_{R}N}e^{-\mu_{R}\theta_{\max}\rho }}{\Gamma \left(m_{R}N\right)}\int_{0}^{\infty }{\left(t+\theta_{\max}\rho \right)}^{m_{R}N-1}e^{-\mu_{R}t}\\ \times \left[1-e^{-\mu_{RU_{1}}a\theta_{\max}\rho }\sum_{a=0}^{N}\left(\begin{array}{c} N\\ a \end{array}\right){\left(-1\right)}^{a}e^{-\frac{\mu_{RU_{1}}a\theta_{\max}\left(\theta_{\max}\rho^{2}+1\right)}{t}}\right.\\ \times \sum_{j_{0}+\ldots +j_{m_{RU_{1}}-1}=a}{\left(\begin{array}{c} a\\ j_{0},\ldots ,j_{m_{RU_{1}}-1} \end{array}\right)\prod_{b=0}^{m_{RU_{1}}-1}\left(\frac{\mu_{RU_{1}}^{b}}{b!}\right)}^{j_{b}}\\ \left.\times {\left(\frac{\theta_{\max}\left(\theta_{\max}\rho^{2}+1\right)}{t}+\theta_{\max}\rho \right)}^{bj_{b}}\right]. \end{array}$$

Using Newton’s binomial, i.e., ${\left(a+x\right)}^{k}=\sum_{v=0}^{k}\left(\begin{array}{c} k\\ v \end{array}\right)x^{v}a^{k-v}$ and Equation (3.351.3) [48], (A8) can be expressed as

(A9) $$\begin{array}{c} {\bar{P}}_{1}^{II}=1-\frac{\mu_{R}^{m_{R}N}e^{-\mu_{R}\theta_{\max}\rho }}{\Gamma \left(m_{R}N\right)}\sum_{q=0}^{m_{R}N-1}\left(\begin{array}{c} m_{R}N-1\\ q \end{array}\right)\\ \times {\left(\theta_{\max}\rho \right)}^{m_{R}N-q-1}\left[\frac{q!}{\mu_{R}^{q+1}}-\sum_{a=0}^{N}\left(\begin{array}{c} N\\ a \end{array}\right){\left(-1\right)}^{a}e^{-\mu_{RU_{1}}a\theta_{\max}\rho }\right.\\ \times \sum_{j_{0}+\ldots +j_{m_{RU_{1}}-1}=a}\left(\begin{array}{c} a\\ j_{0},\ldots ,j_{m_{RU_{1}}-1} \end{array}\right)\prod_{b=0}^{m_{RU_{1}}-1}{\left(\frac{\mu_{RU_{1}}^{b}}{b!}\right)}^{j_{b}}\\ \times \sum_{c=0}^{bj_{b}}\left(\begin{array}{c} bj_{b}\\ c \end{array}\right){\left[\theta_{\max}\left(\theta_{\max}\rho^{2}+1\right)\right]}^{c}{\left(\theta_{\max}\rho \right)}^{bj_{b}-c}\\ \times \int_{0}^{\infty }e^{-\mu_{R}t-\frac{\mu_{RU_{1}}a\theta_{\max}\left(\theta_{\max}\rho^{2}+1\right)}{t}}t^{q-c}dt. \end{array}$$

With the aid of Equation (3.471.9) [48], we have $P_{1}^{II}$, which can be obtained by

(A10) $$\begin{array}{c} {\bar{P}}_{1}^{II}=1-\frac{\mu_{R}^{m_{R}N}e^{-\mu_{R}\theta_{\max}\rho }}{\Gamma \left(m_{R}N\right)}\sum_{q=0}^{m_{R}N-1}\left(\begin{array}{c} m_{R}N-1\\ q \end{array}\right)\\ \times {\left(\theta_{\max}\rho \right)}^{m_{R}N-q-1}\left[\frac{q!}{\mu_{R}^{q+1}}-\sum_{a=0}^{N}\left(\begin{array}{c} N\\ a \end{array}\right){\left(-1\right)}^{a}\right.\\ \times e^{-\mu_{RU_{1}}a\theta_{\max}\rho }\sum_{j_{0}+\ldots +j_{m_{RU_{1}}-1}=a}\left(\begin{array}{c} a\\ j_{0},\ldots ,j_{m_{RU_{1}}-1} \end{array}\right)\\ \times \prod_{b=0}^{m_{RU_{1}}-1}{\left(\frac{\mu_{RU_{1}}^{b}}{b!}\right)}^{j_{b}}\sum_{c=0}^{bj_{b}}\left(\begin{array}{c} bj_{b}\\ c \end{array}\right){\left[\theta_{\max}\left(\theta_{\max}\rho^{2}+1\right)\right]}^{c}\\ \times {\left(\theta_{\max}\rho \right)}^{bj_{b}-c}2{\left(\frac{\mu_{RU_{1}}a\theta_{\max}\left(\theta_{\max}\rho^{2}+1\right)}{\mu_{R}}\right)}^{\frac{q-c+1}{2}}\\ \left.\times K_{q-c+1}\left(2\sqrt{\mu_{R}\mu_{RU_{1}}a\theta_{\max}\left(\theta_{\max}\rho^{2}+1\right)}\right)\right]. \end{array}$$

The proof is completed. □

## Appendix C

**Proof** **of Proposition 4.** Ergodic Capacity of $U_{2}$

(A11) $$\begin{array}{cc} {\bar{C}}_{2}= & E\left\{\frac{1}{2}\log_{2}\left(1+\underset{X}{\underset{︸}{\max\left(\gamma_{U_{2}},\gamma_{RU_{2},x_{2}}\right)}}\right)\right\}\\ = & \frac{1}{2\ln2}\int_{0}^{\frac{a_{2}}{a_{1}}}\frac{1}{1+x}\left[1-F_{X}\left(\frac{x}{a_{2}-a_{1}x}\right)\right]dx \end{array}$$

By the variable changing $t=\frac{x}{a_{2}-a_{1}x}$ and after a few steps, (A11) can then [50]

(A12) $$\begin{array}{c} {\bar{C}}_{2}=\frac{1}{2\ln2}\int_{0}^{\infty }\left(\frac{1}{t+{\left(a_{2}+a_{1}\right)}^{-1}}-\frac{1}{t+a_{1}^{-1}}\right)\left[1-F_{X}\left(t\right)\right]dt. \end{array}$$

From ${\bar{A}}_{1}$ and ${\bar{A}}_{2}$, we have $F_{X}\left(t\right)$ calculated as

(A13) $$\begin{array}{cc} F_{X}\left(t\right)= & \mathrm{Pr}\left(X_{2}<\frac{t}{\rho }\right)\mathrm{Pr}\left(Y_{n}>\frac{t\left(Z_{2n}+\rho^{-1}\right)}{\rho \left(Z_{2n}-\theta \right)},Z_{2n}>\frac{t}{\rho }\right)\\ = & 1-e^{-\frac{\mu_{U_{2}}}{\rho }t}\sum_{a=0}^{m_{U_{2}}-1}\frac{\mu_{U_{2}}^{a}t^{a}}{a!\rho^{a}}-2\left(1-e^{-\frac{\mu_{U_{2}}}{\rho }t}\sum_{a=0}^{m_{U_{2}}-1}\frac{\mu_{U_{2}}^{a}t^{a}}{a!\rho^{a}}\right)\\ \; & \times \sum_{b=0}^{m_{R}N-1}\sum_{q=0}^{b}\sum_{n=0}^{m_{RU_{2}}N-1}\left(\begin{array}{c} b\\ q \end{array}\right)\left(\begin{array}{c} m_{RU_{2}}N-1\\ n \end{array}\right)\\ \; & \times \frac{\mu_{RU_{2}}^{m_{RU_{2}}N}\mu_{R}^{b}}{b!\Gamma \left(m_{RU_{2}}N\right)}{\left(\frac{t^{2}}{\rho^{2}}+t\right)}^{q}e^{-\theta \left(\mu_{RU_{2}}+\mu_{R}\right)}\\ \; & \times {\left(\frac{t}{\rho }\right)}^{m_{RU_{2}}N+b-q-n-1}{\left(\frac{\mu_{R}}{\mu_{RU_{2}}}\left(\frac{t^{2}}{\rho^{2}}+t\right)\right)}^{\frac{n-q+1}{2}}\\ \; & \times K_{n-q+1}\left(2\sqrt{\mu_{R}\mu_{RU_{2}}\left(\frac{t^{2}}{\rho^{2}}+t\right)}\right). \end{array}$$

Substituting (A13) into (A11), ${\bar{C}}_{2}$ is given by

(A14) $${\bar{C}}_{2}=\frac{1}{2\ln2}\left({\bar{D}}_{1}+{\bar{D}}_{2}\right),$$

where

(A15) $$\begin{array}{cc} {\bar{D}}_{1}= & \sum_{a=0}^{m_{U_{2}}-1}\frac{\mu_{U_{2}}^{a}}{a!\rho^{a}}\int_{0}^{\infty }\left(\frac{1}{t+{\left(a_{2}+a_{1}\right)}^{-1}}-\frac{1}{t+a_{1}^{-1}}\right)t^{a}e^{-\frac{\mu_{U_{2}}}{\rho }t}dt\\ = & \sum_{a=0}^{m_{U_{2}}-1}\frac{\mu_{U_{2}}^{a}}{a!\rho^{a}}\left[\int_{0}^{\infty }\frac{t^{a}e^{-\frac{\mu_{U_{2}}}{\rho }t}}{t+{\left(a_{2}+a_{1}\right)}^{-1}}dt-\int_{0}^{\infty }\frac{t^{a}e^{-\frac{\mu_{U_{2}}}{\rho }t}}{t+a_{1}^{-1}}dt\right]. \end{array}$$

It is worth noting that $e^{-ax}$ can be expressed in terms of the Meijer-G function as Equation (40) [51]

(A16) $$e^{-ax}=G_{0,1}^{1,0}\left(ax\left|\begin{array}{c} -\\ 0 \end{array}\right.\right),$$

where $G_{p,q}^{m,n}\left[.\right]$ Equation (9.301) [48] is the Meijer G-function, and (A15) is rewritten as

(A17) $$\begin{array}{cc} {\bar{D}}_{1}= & \sum_{a=0}^{m_{U_{2}}-1}\frac{\mu_{U_{2}}^{a}}{a!\rho^{a}}\left[\int_{0}^{\infty }\frac{t^{a}}{t+{\left(a_{2}+a_{1}\right)}^{-1}}G_{0,1}^{1,0}\left(\frac{\mu_{U_{2}}}{\rho }t\left|\begin{array}{c} -\\ 0 \end{array}\right.\right)dt\right.\\ \; & \left.-\int_{0}^{\infty }\frac{t^{a}}{t+a_{1}^{-1}}G_{0,1}^{1,0}\left(\frac{\mu_{U_{2}}}{\rho }t\left|\begin{array}{c} -\\ 0 \end{array}\right.\right)dt\right]. \end{array}$$

Applying Equation (7.811.5) [48], ${\bar{D}}_{1}$ can be obtained as follows

(A18) $$\begin{array}{c} {\bar{D}}_{1}=\sum_{a=0}^{m_{U_{2}}-1}\frac{\mu_{U_{2}}^{a}}{a!\rho^{a}}\left[\frac{1}{{\left(a_{2}+a_{1}\right)}^{a}}G_{1,2}^{2,1}\left(\frac{\mu_{U_{2}}}{\rho \left(a_{2}+a_{1}\right)}\left|\begin{array}{c} 1-a-1,-\\ 1-a-1,0 \end{array}\right.\right)\right.\\ \;\;\;\;\;\;\;\;\;\;\;\;\;\;\;\left.-\frac{1}{a_{1}^{a}}G_{1,2}^{2,1}\left(\frac{\mu_{U_{2}}}{\rho a_{1}^{a}}\left|\begin{array}{c} 1-a-1,-\\ 1-a-1,0 \end{array}\right.\right)\right]. \end{array}$$

Next, ${\bar{D}}_{2}$ can be calculated by

(A19) $$\begin{array}{cc} \; & {\bar{D}}_{2}=2\sum_{b=0}^{m_{R}N-1}\sum_{q=0}^{b}\sum_{n=0}^{m_{RU_{2}}N-1}\left(\begin{array}{c} b\\ q \end{array}\right)\left(\begin{array}{c} m_{RU_{2}}N-1\\ n \end{array}\right)\\ \; & \times \frac{\mu_{RU_{2}}^{m_{RU_{2}}N}\mu_{R}^{b}}{b!\Gamma \left(m_{RU_{2}}N\right)}\int_{0}^{\infty }\left(\frac{1}{t+{\left(a_{2}+a_{1}\right)}^{-1}}-\frac{1}{t+a_{1}^{-1}}\right)\\ \; & \times {\left(\frac{\mu_{R}}{\mu_{RU_{2}}}\left(\frac{t^{2}}{\rho^{2}}+\frac{t}{\rho^{2}}\right)\right)}^{\frac{n-q+1}{2}}\left(1-e^{-\frac{\mu_{U_{2}}}{\rho }t}\sum_{a=0}^{m_{U_{2}}-1}\frac{\mu_{U_{2}}^{a}t^{a}}{a!\rho^{a}}\right)\\ \; & \times {\left(\frac{t^{2}}{\rho^{2}}+\frac{t}{\rho^{2}}\right)}^{q}e^{-\frac{t}{\rho }\left(\mu_{RU_{2}}+\mu_{R}\right)}{\left(\frac{t}{\rho }\right)}^{m_{RU_{2}}N+b-q-n-1}\\ \; & \times K_{n-q+1}\left(2\sqrt{\mu_{R}\mu_{RU_{2}}\left(\frac{t^{2}}{\rho^{2}}+\frac{t}{\rho^{2}}\right)}\right)dt. \end{array}$$

Let $r=\frac{4}{\pi }\arctan\left(t\right)-1\Rightarrow \tan\left(\frac{\pi \left(r+1\right)}{4}\right)=t\Rightarrow \frac{\pi }{4}\sec^{2}\left(\frac{\pi }{4}\left(r+1\right)\right)dr=dt$; then, (A18) is given by

(A20) $$\begin{array}{cc} \; & {\bar{D}}_{2}=\frac{\pi }{2}\sum_{b=0}^{m_{R}N-1}\sum_{q=0}^{b}\sum_{n=0}^{m_{RU_{2}}N-1}\left(\begin{array}{c} b\\ q \end{array}\right)\left(\begin{array}{c} m_{RU_{2}}N-1\\ n \end{array}\right)\\ \; & \times \frac{\mu_{RU_{2}}^{m_{RU_{2}}N}\mu_{R}^{b}}{b!\Gamma \left(m_{RU_{2}}N\right)}\int_{-1}^{1}\left(\frac{1}{\Lambda \left(r\right)+{\left(a_{2}+a_{1}\right)}^{-1}}-\frac{1}{\Lambda \left(r\right)+a_{1}^{-1}}\right)\\ \; & \times \sec^{2}\left(\frac{\pi }{4}\left(r+1\right)\right)\left(1-e^{-\frac{\mu_{U_{2}}\Lambda \left(r\right)}{\rho }}\sum_{a=0}^{m_{U_{2}}-1}\frac{\mu_{U_{2}}^{a}\Lambda {\left(r\right)}^{a}}{a!\rho^{a}}\right)\\ \; & \times \Phi {\left(r\right)}^{q}e^{-\frac{\Lambda \left(r\right)}{\rho }\left(\mu_{RU_{2}}+\mu_{R}\right)}{\left(\frac{\Lambda \left(r\right)}{\rho }\right)}^{m_{RU_{2}}N+b-q-n-1}\\ \; & \times {\left(\frac{\mu_{R}\Phi \left(r\right)}{\mu_{RU_{2}}}\right)}^{\frac{n-q+1}{2}}K_{n-q+1}\left(2\sqrt{\mu_{R}\mu_{RU_{2}}\Phi \left(r\right)}\right). \end{array}$$

Unfortunately, finding a closed-form expression for (A19) is a tough task, but an accurate approximation can be obtained for it. By using the Gaussian–Chebyshev quadrature shown in Equation (25.4.38) [49], it can be achieved by

(A21) $$\begin{array}{cc} {\bar{D}}_{2}\approx & \frac{\pi^{2}}{2D}\sum_{b=0}^{m_{R}N-1}\sum_{q=0}^{b}\sum_{n=0}^{m_{RU_{2}}N-1}\sum_{d=1}^{D}\left(\begin{array}{c} b\\ q \end{array}\right)\left(\begin{array}{c} m_{RU_{2}}N-1\\ n \end{array}\right)\\ \; & \times \frac{\mu_{RU_{2}}^{m_{RU_{2}}N}\mu_{R}^{b}}{b!\Gamma \left(m_{RU_{2}}N\right)}\sqrt{1-\varphi_{d}^{2}}\sec^{2}\left(\frac{\pi }{4}\left(\varphi_{d}+1\right)\right)\\ \; & \times \left(\frac{1}{\Lambda \left(\varphi_{d}\right)+{\left(a_{2}+a_{1}\right)}^{-1}}-\frac{1}{\Lambda \left(\varphi_{d}\right)+a_{1}^{-1}}\right)\\ \; & \times \left(1-e^{-\frac{\mu_{U_{2}}\Lambda \left(\varphi_{d}\right)}{\rho }}\sum_{a=0}^{m_{U_{2}}-1}\frac{\mu_{U_{2}}^{a}\Lambda {\left(\varphi_{d}\right)}^{a}}{a!\rho^{a}}\right)\\ \; & \times \Phi {\left(\varphi_{d}\right)}^{q}e^{-\frac{\Lambda \left(\varphi_{d}\right)}{\rho }\left(\mu_{RU_{2}}+\mu_{R}\right)}{\left(\frac{\Lambda \left(\varphi_{d}\right)}{\rho }\right)}^{m_{RU_{2}}N+b-q-n-1}\\ \; & \times {\left(\frac{\mu_{R}\Phi \left(\varphi_{d}\right)}{\mu_{RU_{2}}}\right)}^{\frac{n-q+1}{2}}K_{n-q+1}\left(2\sqrt{\mu_{R}\mu_{RU_{2}}\Phi \left(\varphi_{d}\right)}\right), \end{array}$$

where $\varphi_{d}=\cos\left(\frac{2d-1}{2D}\pi \right)$, $\Lambda \left(r\right)=\tan\left(\frac{\pi \left(r+1\right)}{4}\right)$ and $\Phi \left(r\right)=\frac{\Lambda \left(r\right)}{\rho^{2}}\left(\Lambda \left(r\right)+1\right)$. The proof is completed. □
